# Supplementary material for: From nanohole to ultralong straight nanochannel fabrication in graphene oxide with swift heavy ions
Source: Nat Commun. 2023 Feb 16;14:889. doi: 10.1038/s41467-023-36357-8 (PMC9935919; doi:10.1038/s41467-023-36357-8)
Supplement: Supplementary file 1 — Supplementary Information [file 41467_2023_36357_MOESM1_ESM.pdf]

Supplementary Information

From nanohole to ultralong straight nanochannel fabrication in graphene oxide with swift heavy ions

Andrzej Olejniczak[1,2,*] & Ruslan A. Rymzhanov[2,3]

1) Faculty of Chemistry, Nicolaus Copernicus University, ul. Gagarina 7, 87-100, Torun, Poland;
2) Joint Institute for Nuclear Research, Joliot-Curie 6, 141980 Dubna, Moscow Region, Russia;
3) Institute of Nuclear Physics, Ibragimov St. 1, 050032 Almaty, Kazakhstan;
* Correspondence to: aolejnic@chem.umk.pl

Supplementary Note 1: Possible applications of the nanoperforated GO

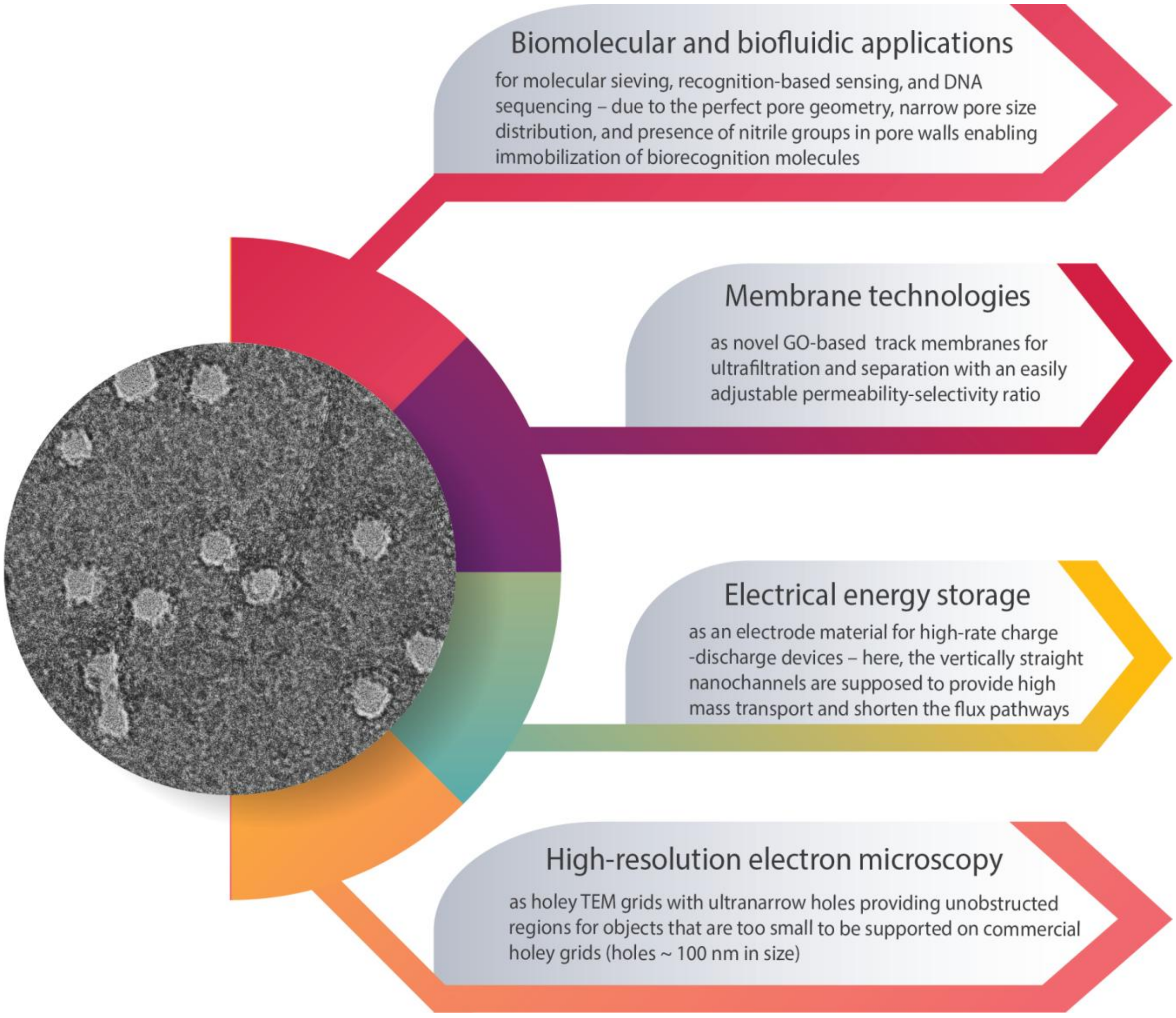

Supplementary Note 2: Calculation of pore geometry parameters from electron microscopy images

Since the contrast in most of our images is rather low, the determination of pore parameters from top-view images is based on the radial intensity profiles, and that from cross-sectional images is based on the aligning and averaging of line profiles.

Calculation of radial intensity profiles

The radial (i.e., angularly averaged) profiles for features in the top-view TEM and SEM images were calculated as follows. For each nanohole (or nanohillock), the central point of the profile was chosen manually, and a preliminary radial profile was calculated. The profile was then optimized (i.e., symmetrized) by: (i) shifting the central point in the x and y (i.e., horizontal and vertical) directions inside a circular region several pixels in size; (ii) calculating the differences in intensities between points at the same radial distance from the center, but in different directions; and (iii) choosing the position for which the sum of weighted differences is lowest.

Processing of radial profiles extracted from TEM images

Exemplary radial profiles extracted from TEM images of suspended few-layer GO irradiated with 710 MeV Bi ions are shown in Supplementary Fig. 1.

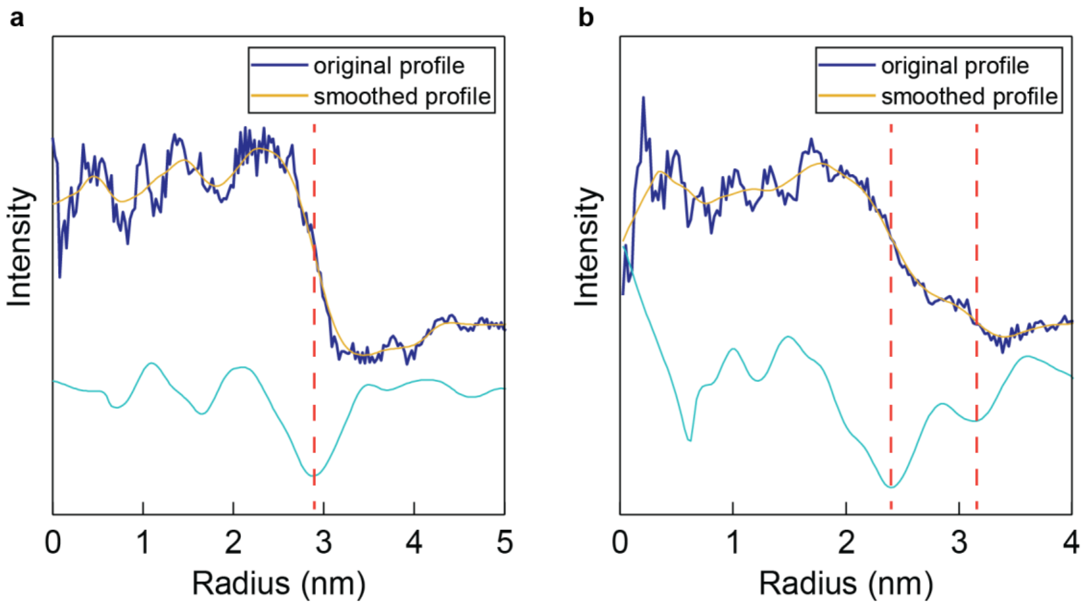

Supplementary Fig. 1: Exemplary radial profiles extracted from TEM images. a Perfectly round-shaped nanopore and **b** nanopore with edge crack. Since the radial intensity drops sharply at the nanohole edge, the 1st derivative method was used to calculate the pore radius.

The profiles were smoothed using the 'Lowess' (locally weighted scatterplot smoothing) algorithm as implemented in MATLAB. For regularly shaped pores (Supplementary Fig. 1a), the radius of the pore was taken as the position of a minimum in the 1st derivative of the smoothed profile. In the case of imperfectly round-shaped nanoholes and those with cracks (single-layer GO case), the irregularities are reflected in the radial profiles as additional intensity drops (Supplementary Fig. 1b); consequently, the corresponding 1st derivative has more than one minimum. To account for this shape-related pore nonuniformity, all the minima were used to create the pore-size histograms.

Processing of radial profiles extracted from SEM images

Example radial profiles of the features created on the surface of GO films with high-S_e ions are shown in Supplementary Fig. 2.

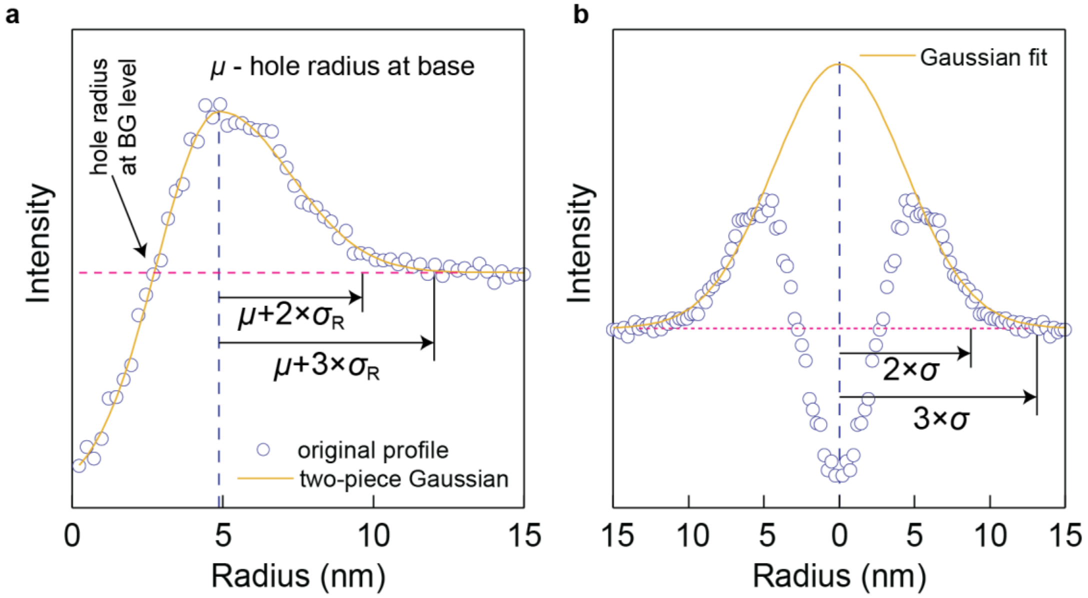

Supplementary Fig. 2: Exemplary radial profile extracted from an SEM image. a Four-parameter two-piece Gaussian fit. **b** Single Gaussian fit to the hillock-like (outer) part of the profile. Horizontal dashed lines represent the signal intensity at the background (BG) level.

First, we subtracted the average signal intensity at the background level, i.e., that of the unmodified part of the GO film in the proximity of the nanohole, estimated by averaging several points at the end of the radial profile. For high-S_e ions, the profiles of crater-like features were fitted with a four-parameter two-piece Gaussian function:

$$\mathrm{Rp}(r) = \begin{cases} \mathrm{norm}\left\{\exp\left[-\frac{(r-\mu)^2}{2\times\sigma_L^2}\right] \times (h_R + h_L) - h_L\right\}, & r \le \mu \\ \mathrm{norm}\left\{\exp\left[-\frac{(r-\mu)^2}{2\times\sigma_R^2}\right] \times h_R\right\}, & r > \mu \end{cases} \tag{S1}$$

where σ_L and σ_R are the Gaussian root mean square widths of the left and right pieces of the curve, respectively; h_L and h_R are the heights of the left and right pieces of the curve, respectively; and μ is the position of the maximum of the two-piece Gaussian function.

From the model, we calculated the following parameters:

- the size of the holes/pores at the background level, i.e., at the point at the inner pore edge with the same signal intensity as the unmodified GO film in proximity,
- the size of the nanopore entry, i.e., the broadest part of the nanopore at its base, assumed to be equal to $2\times\mu$.
- the size of the nanohillock, taken as $2(\mu + 3\times\sigma_R)$

Alternatively, the sizes of the nanohillocks were taken as $2\times3\sigma$ of a single Gaussian fitted to the hillock-like part (i.e., the outer slope) of the background subtracted profile. Both estimations yield closely similar values (Supplementary Fig. 2a and b).

A similar approach to hillock size determination was undertaken for low-S_e ions that do not create nanoholes. Here, the Gaussian function was fitted to the whole background subtracted profiles, and the sizes of the hillocks were taken as $2\times3\sigma$.

Note that the width of 3σ accounts for 99.73% of the area under the Gaussian curve. The values can easily be recalculated to a 2σ width, which closely matches the sizes of features that one would obtain by manually marking the edges on the image (i.e., with subjective naked eye criterion).

Extraction of averaged line profiles from cross-sectional SEM images

To estimate the width of the nanochannels from the cross-sectional SEM images, we used the following procedure. Initially, several line profiles from the top and bottom part of the nanochannel were extracted and averaged separately. The averaged profiles were then aligned by rotating the image so that the intensity minima representing the nanochannel center match each other. Finally, the line profiles were extracted and averaged over the whole nanochannel length visible in the aligned image.

Supplementary Note 3: Compositional parameters used in MC-RxMD simulations

Throughout the section "MC-RxMD modeling of pore formation and structural recovery", we use the following notation:

C_{tot} – total number of C atoms in the supercell;

C_{rem} – number of C atoms removed from the GO lattice;

C_{all} – number of C atoms bonded to the GO lattice;

C_{sub} – number of substituted C atoms, i.e., bonded to at least one heteroatom;

C_{unsub} – number of unsubstituted C atoms, i.e., bonded only to other C atom(s);

C_{rem}/C_{tot} – reflects the removal efficiency of C atoms, i.e., the fraction of C atoms lost from the GO lattice during cell relaxation or due to ion passage;

C_{sub}/C_{all} – substitution degree; alternatively, the substitution degree is expressed by C_{sub}/C_{unsub};

$\Delta(C_{sub}/C_{all})$ – absolute difference in the substitution degree (C_{sub}/C_{all}) between relaxed and irradiated cells, i.e., $\Delta(C_{sub}/C_{all}) = (C_{sub}^{relax}/C_{all}^{relax} - C_{sub}^{irrad}/C_{all}^{irrad})$;

$\Delta(C_{sub}/C_{all})/(C_{sub}^{relax}/C_{all}^{relax})$ – relative difference in the substitution degree between relaxed and irradiated cells; obtained by dividing $\Delta(C_{sub}/C_{all})$ by the substitution degree for the relaxed cell $(C_{sub}^{relax}/C_{all}^{relax})$;

$C_{sub}/C_{all}^{\#}$ – plotted on the radial distribution profiles (Fig. 5b and Supplementary Fig. 15b); represents the local ratio of substitution degree $C_{n,sub}^{irrad}/C_{n,all}^{irrad}$ in the n-th cylindrical layer of the irradiated cell to $C_{sub}^{relax}/C_{all}^{relax}$ averaged over all layers for the relaxed cell;

$\Delta(C_{sub}/C_{all})$, $\Delta(C_{sub}/C_{all})/(C_{sub}^{relax}/C_{all}^{relax})$, and $C_{sub}/C_{all}^{\#}$ are used to describe the extent of defunctionalization due to ion passage.

The parameters for initial, relaxed, and ion-irradiated cells are denoted with the superscripts 'init', 'relax', and 'irrad', respectively.

Supplementary Note 4: Remarks on GO synthesis and flake sizes

Procedure for flake size determination

The flake size distributions were determined from SEM images. For imaging, the specimens were prepared by drop casting the diluted water–GO suspensions onto precleaned Si wafers. For each specimen, several SEM images were recorded at an acceleration voltage of 2 kV and with an SE detector. The sizes of the flakes were measured manually in two perpendicular directions for each flake; only isolated flakes were taken into account.

As is known, the appearance of the histogram can be strongly influenced by the number of bins and their centers [1]. Here, in the size distribution histograms, the number of bins N_b = ceil((x_{max}-x_{min})/h) was chosen to be slightly lower than that resulting from the Scott criterion $h = 3.49 \times SD \times N^{-1/3}$, where h is the optimal bin width, SD is the standard deviation and N denotes the number of determinations [2, 3]. The relevance of this approach was inspected by comparing the obtained histograms with the respective kernel densities estimated using the Gaussian kernel (Supplementary Fig. 3b and 4b).

For both samples (GO-M and GO-Z), the flake size distributions were fairly well fitted with a log-normal distribution:

$$\mathrm{PDF}(x) = \frac{1}{x\sigma\sqrt{2\pi}}\exp\left(-\frac{(\ln(x)-\mu)^2}{2\sigma^2}\right) \quad \text{(S2)}$$

where μ and σ are the shape and location parameters of the log-normal distribution, respectively.

The mode, mean ($\bar{x}$), and standard deviation (SD) of the log-normal distribution were calculated as follows:

$$\mathrm{mode} = \exp(\mu - \sigma^2) \quad \text{(S3)}$$

$$\bar{x} = \exp(\mu + \sigma^2/2) \quad \text{(S4)}$$

$$\mathrm{SD} = \bar{x}\sqrt{\exp(\sigma^2) - 1} \quad \text{(S5)}$$

A log-normal distribution was previously reported for some 2D materials and is characteristic of the random fragmentation of flakes with a random distribution of the sizes and shapes of the resulting pieces [3].

Synthesis and flake size distribution of the GO-Z sample

GO-Z was synthesized as follows. Flake graphite (2 g) was added to ice-cooled concentrated sulfuric acid (150 ml) and stirred for a few minutes. Then, sodium nitrite (1.5 g) was added, the mixture was stirred for 5 minutes, and potassium permanganate (9 g) was added slowly. The mixture was stirred for 2 h in ice bath; then, stirring was continued at room temperature for a period of time chosen so that the desired C/O ratio of the final GO-Z films closely matched that of the GO-M films (5 days in this case). After that, 5% sulfuric acid (280 ml) was added slowly, and the mixture was kept for 2 h at 98 °C under stirring. After the mixture cooled to 60 °C, 30% hydrogen peroxide was added, and stirring was continued for another

2 h at room temperature. Then, the reaction mixture was purified by extensive washing (several times) with a mixture of sulfuric acid (3%)/hydrogen peroxide (0.5%), then with 3% hydrochloric acid and subsequently with distilled water. Final purification was carried out by dialyzing the water suspension of the resulting GO slurry for several days.

A representative SEM image of GO-Z along with a histogram of flake sizes is shown in Supplementary Fig. 3. The majority of GO-Z flakes are ca. 200-500 nm in size, small flakes of 50-100 nm are quite frequent, and the largest flakes typically do not exceed 1.5 μm. The flake size distribution is fairly well described by the log-normal distribution function with a mode of 190 nm, a mean value of 350 nm and a standard deviation of ~ 250 nm. These results are in good agreement with previous AFM measurements of GO-Z [4] and with Zhang et al. [5,] who claimed that their procedure produces rather small GO flakes that have an average diameter of 212 nm and a Gaussian distribution of areas with a maximum centered at 0.02 μm^2. AFM investigations of GO-Z carried out by Mravik et al. [4] showed that the thickness of most of the flakes is ~ 1.1-1.4 nm, consistent with the apparent height of the monolayer GO sheet (~ 1.1±0.3 nm) [6]. The high content of monolayer flakes is also confirmed by their light gray color in the SEM images (Supplementary Fig. 3a).

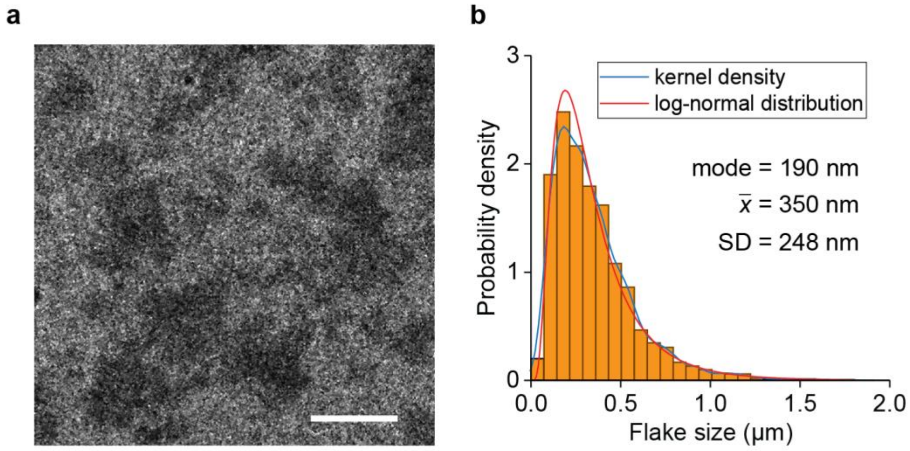

Supplementary Fig. 3: Flake size distribution of the GO-Z sample. a SEM image; the scale bar is 0.5 μm. **b** Histogram of flake size distribution along with kernel density (Gaussian kernel, 0.04 μm binwidth) and log-normal distribution fit. Source data are provided as a Source Data file.

Synthesis and flake size distribution of the GO-Z sample

To obtain GO-M, a mixture of flake graphite, concentrated sulfuric acid and potassium permanganate was mixed with a high-shear mixer for 1 h. After the oxidation reaction was finished and the slurry was extensively washed to remove inorganic contaminants, it was diluted with water and subjected to additional high-shear mixing to increase the extent of exfoliation.

An SEM image of the GO-M specimen along with the corresponding flake size distribution is shown in Supplementary Fig. 4. Here, the majority of flakes are 0.5-1.5 μm in size, and the large flakes rich sizes of 4-6 μm. The flake size distribution follows a log-normal distribution with a mode of ~0.89 μm, a mean

value of ~1.32 µm and a standard deviation of ~0.74 µm. This result is consistent with the fact that high-shear mixing facilitates exfoliation of graphene and GO and allows large-area flakes to be obtained [7]. The deviation from the log-normal distribution, i.e., a higher content of large flakes at the cost of intermediate flakes, can be attributed to the occurrence of both bulk and edge fragmentation [3]. Based on SEM imaging, the content of single-layer flakes was estimated to be > 90%.

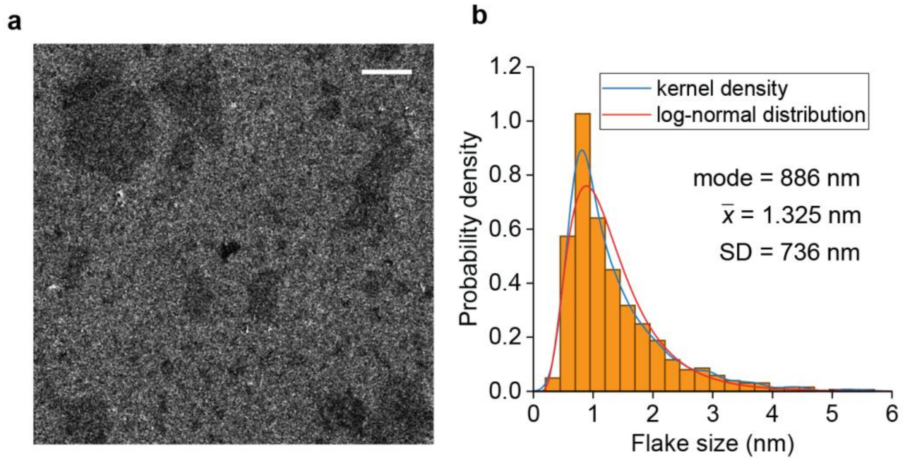

Supplementary Fig. 4: Flake size distribution of the GO-M sample. a SEM image; the scale bar is 2 µm. **b** Histogram of flake size distribution along with kernel density (Gaussian kernel, 0.15 µm binwidth) and log-normal distribution fit. Individual multilayer GO flakes are represented by a darker gray color and are rare; the overlapped flakes are possibly produced by flake agglomeration during sample deposition [8]. Source data are provided as a Source Data file.

Supplementary Note 5: MC-RxMD modeling of SHI irradiation damage and structural recovery

MC simulations of excitation of the electronic subsystem

Simulations of excitation of the electronic subsystem and initial lattice excitation were carried out using MC TREKIS code that takes into account the following processes:

- penetration of a swift heavy projectile resulting in ionization of target electrons and creation of primary electrons (δ-electrons) and holes;
- scattering of δ-electrons by lattice atoms and target electrons;
- kinetics of the generation of SEs arising during relaxation of the electronic subsystem;
- Auger decay of core holes, resulting in the production of SEs;
- radiative decay of core holes, subsequent photon transport, and excitation of new electrons and holes by photoabsorption;
- spatial redistribution of valence holes.

The cross sections used in TREKIS take into account the collective responses of the electronic and atomic subsystems of a target in the framework of the dynamic structure factor formalism. This approach, within the first Born approximation, links the loss function, defined as the inverse imaginary part of the complex dielectric function (CDF, $\varepsilon(\omega,q)$) with the cross section of scattering of an incident particle on a spatially and dynamically coupled system of scattering centers [9]:

$$\frac{\mathrm{d}^2\sigma}{\mathrm{d}(\hbar\omega)\mathrm{d}(\hbar q)} = \frac{2(Z_\mathrm{e}(v)e)^2}{n_\mathrm{sc}\pi\hbar^2v^2}\frac{1}{\hbar q}\left[1-\mathrm{e}^{-\frac{\hbar\omega}{k_\mathrm{B}T}}\right]^{-1}\mathrm{Im}\left[\frac{-1}{\varepsilon(\omega,q)}\right] \quad \text{(S6)}$$

where $\hbar\omega$ is the transferred energy, $\hbar q$ is the transferred momentum ($\hbar$ is the Planck constant), Z_e is the effective charge of the incident particle [10, 11], e is the electron charge, v is the velocity of the particle, n_sc is the density of scattering centers, equal to the atomic or molecular density (depending on the normalization of the CDF), T is the temperature of the sample (room temperature in our case) and k_B is the Boltzmann constant.

According to the algorithm proposed by Ritchie and Howie, the loss function can be reconstructed from experimental (or calculated) optical data on photon scattering on solid [12]:

$$\mathrm{Im}\left[\frac{-1}{\varepsilon(\omega,q=0)}\right] = \sum_{i=1}^{N^\mathrm{os}}\frac{A_\mathrm{i}\gamma_\mathrm{i}\hbar\omega}{\left[\hbar^2\omega^2-E_{0\mathrm{i}}^2(q=0)\right]^2+(\gamma_\mathrm{i}\hbar\omega)^2} \quad \text{(S7)}$$

where $E_{0\mathrm{i}}$ is the characteristic energy of the i-th oscillator, A_i is the fraction of electrons with energy $E_{0\mathrm{i}}$, and γ_i is the i-th energy damping coefficient.

Since the experimental optical data of GO is available only in a limited photon energy range (<6 eV; see, e.g., [13, 14]), the high-energy branch of the loss function (>30 eV) was calculated according to Henke et al. [15, 16] using the actual elemental composition of modeled GO. In the low energy region (<30 eV), we found a significant disagreement between the published data [13, 14] (Supplementary Fig. 5). Because of this and the lack of experimental data in the important region of 6-30 eV, we used the optical constants of

graphite taken from [15, 17]. Note that the data points for graphite lie between those reported for GO (see Supplementary Fig. 5). The interaction of excited electrons with the target lattice was taken into account using Mott atomic cross-sections with a modified Molier's screening parameter [18, 19]. Supplementary Table 3 lists the coefficients of the energy loss function of GO in the form of optical oscillators.

MC calculations allowed us to obtain temporal dependencies of the radial distributions of the density and energy of electrons as well as holes in the valence band and different atomic shells and the energy transferred into the atomic system of a target [20]. To obtain reliable statistics, the MC procedure was iterated ~10^3 times [10, 11].

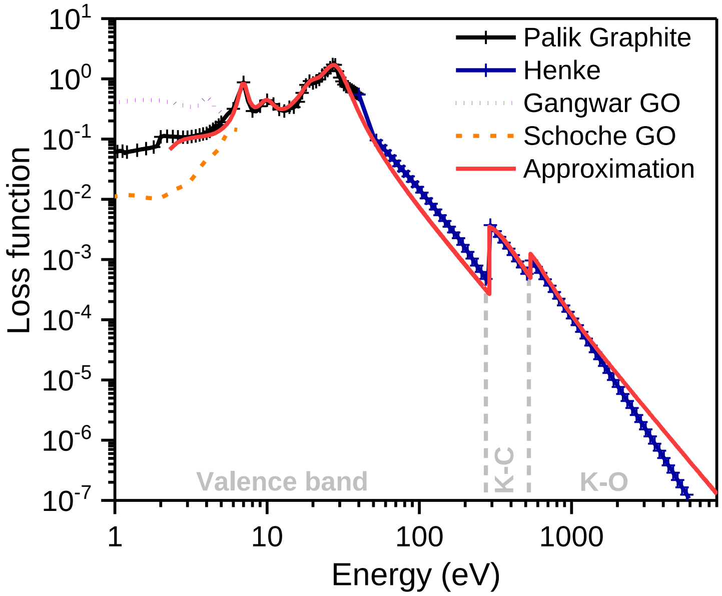

Supplementary Fig. 5: Experimental loss function of graphite (Palik [17]); experimental (Gangwar [13], Schöche [14]) and calculated (Henke [15]) loss functions of GO. Vertical dashed lines mark the different atomic levels in different elements according to the ionization potentials. Source data are provided as a Source Data file.

Using the MC-derived initial energy deposited into the lattice (Supplementary Fig. 6), we set the velocities of atoms in cylindrical layers around the SHI trajectory, assuming a Gaussian-like dispersion of their kinetic energy and a uniform distribution of the momenta of atoms within each layer [20]. This distribution of velocities is used as an initial condition to simulate the lattice response within the MD approach employing the ReaxFF reactive force field in LAMMPS [21]. As shown, the ReaxFF potential is capable of reproducing the majority of reaction mechanisms occurring during the bombardment of graphene with hyperthermal O atoms [22].

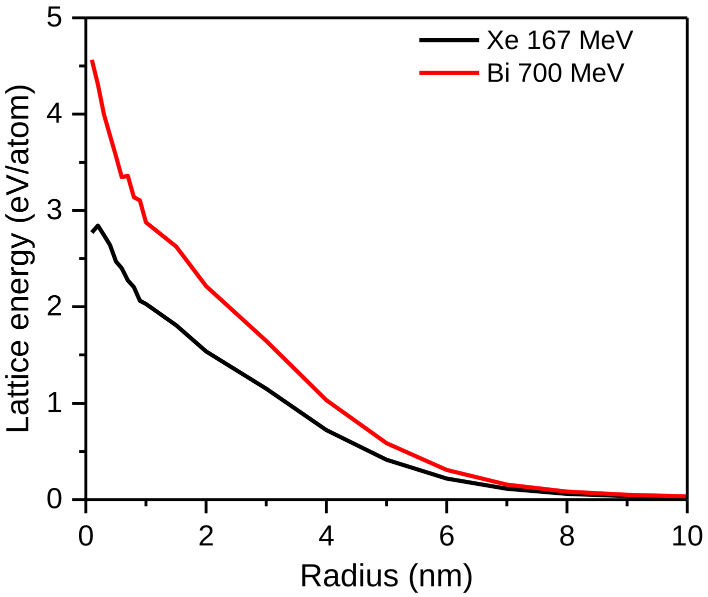

Supplementary Fig. 6: Energy transferred to the atomic subsystem of GO at 100 fs after ion impact. Source data are provided as a Source Data file.

Construction of GO models

GO supercells were constructed based on a graphene sheet consisting of 33120 C atoms (~ 29.4×29.3 nm^2). Structures with the required numbers of oxygen functional groups (denoted 'initial', Table 1) obeying the following rules were generated. First, epoxy groups were distributed at random ring positions and with random top/bottom orientation. This was done by placing O atoms above or below the respective C-C bond and displacing C atoms so that the bond lengths and angles within close proximity of the epoxy group corresponded to density functional theory (DFT) calculations (Supplementary Fig. 7). Then, the required number of hydroxyl groups were distributed among unsubstituted C atoms, with random top/bottom orientation.

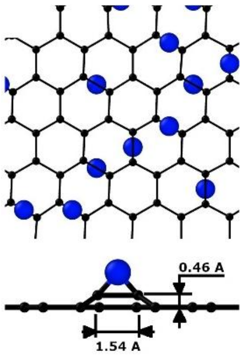

Supplementary Fig. 7: Initial distribution of epoxy groups along with displacements of adjacent C atoms.

A multilayer GO structure, consisting of 403 200 atoms, was constructed by combining a single GO sheet with semirandomly distributed epoxy/OH groups at an initial intersheet distance of 8 Å. After relaxation of the multilayer structure, the average distance between GO sheets was estimated to be ~6.6 Å. This self-assembly was driven by interplane interactions, mainly hydrogen bonding interactions. No artificial stabilization of the modeled structure in the simulation box was applied.

Initial equilibration of GO supercells

All GO supercells were relaxed in the NPT ensemble by heating from 10 to 300 K at a rate of 10^{-3} K/timestep followed by 100 ps equilibration at 300 K. The timestep was 0.25 fs. The structures obtained at this stage are named ‘relaxed’, and their compositions are listed in Table 1 and Supplementary Table 2. Note that after the equilibration, in addition to partial defunctionalization (as discussed in the main text), elongation and cleavage of the C-C bonds of some epoxide rings also occur. This effect is in agreement with DFT calculations showing that if more than one epoxy O atom is attached to a hexagonal C ring, then the epoxy groups in some configurations (in particular, so-called epoxy chains [23]) produce tension sufficient to cleave the underlying C-C bonds [24, 25]. Since in our RxMD simulations, the bond graph is traced down to the bond order (BO) cutoff of 0.3, corresponding to a C-C bond length (BL) of 2 Å, these separated C atoms in the BO and BL histograms are represented as being bonded with weak bonds (Supplementary Figs. 23 and 24).

Modeling of the structural transformations of GO induced by heavy ion impacts

MD simulations of ion impacts were carried out using relaxed supercells with periodic boundary conditions in the lateral directions and open surfaces perpendicular to the ion trajectory. To model a free (open) surface, the box boundaries were extended by 10 nm in both directions along the Z axis. The lateral borders of the cells, 0.5 nm in thickness, were cooled using the Berendsen thermostat with a temperature set to 300 K and a time constant of 0.1 ps [26]. After projectile passage, the structural evolution was traced until 0.1 ns. This time was sufficient for the temperature to drop below 400 K and for the pore size to stabilize.

Supplementary figures

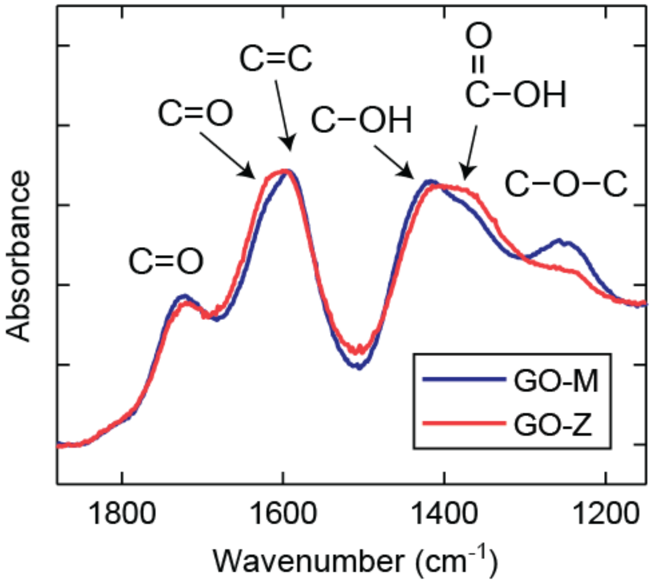

Supplementary Fig. 8: Overlaid FT-IR spectra of pristine GO-Z and GO-M films in the low wavenumber range (1700-1150 cm^{-1}). The spectra were normalized to the same intensity of the ~1600 cm^{-1} signal representing C=C vibrations of the *sp*^{2} carbon network. The first C=O band (1720 cm^{-1}) is ascribed to several carbonyl-bearing moieties, whereas the second C=O mode (~1615 cm^{-1}) is typically attributed to carboxyl groups [27]. The bending C-OH modes span from 1310-1455 cm^{-1}, with the high-wavenumber component (1420 cm^{-1}) ascribed to hydroxyl groups attached to the carbon honeycomb and the low-wavenumber component (1380 cm^{-1}) attributed to C-OH bending vibrations in carboxyl groups. The band C-O-C (1250 cm^{-1}) is due to C-O stretching vibrations in epoxy groups.

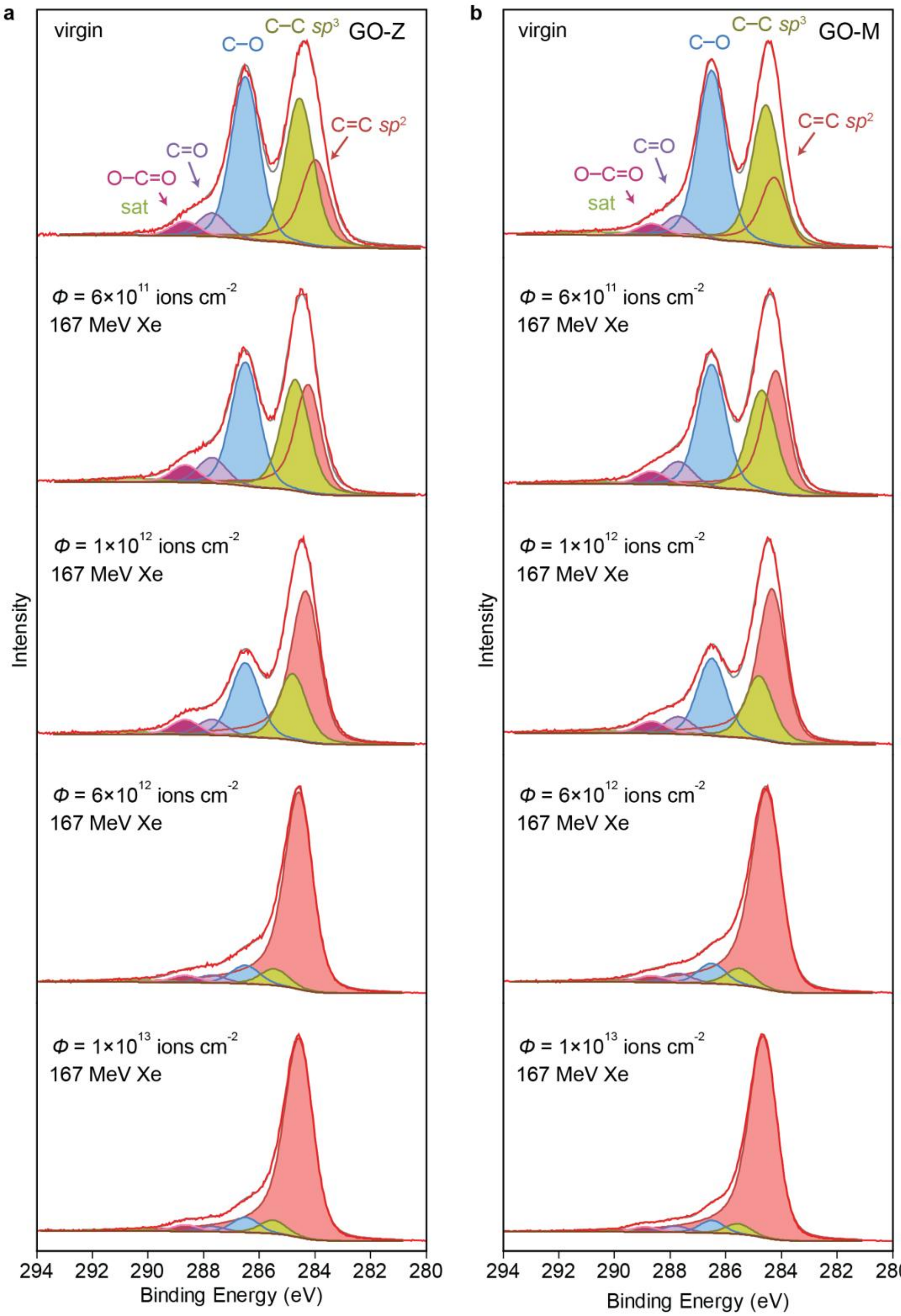

Supplementary Fig. 9: Evolution of the C 1*s* spectra of GO films irradiated with different fluences of 167 MeV Xe ions. a GO-Z films. **b** GO-M films. The spectra were deconvoluted with 6 components centered at ca. 284.5 eV (C=C sp^2), 285.0 eV (C-C sp^3), 286.5 eV (C-O), 287.7 eV (C=O), 288.7 eV (O-C=O), and 290.3 eV (sat). Here, C=C denotes sp^2 graphitic carbon, C-C is attributed to sp^3-hybridized carbon, C-O is due to hydroxyl and epoxy groups, C=O is ascribed to different carbonyl-bearing moieties, O-C=O is mainly due to carboxyl groups, and sat is a π-π* shake-up satellite. The asymmetric peak shape of the C=C sp^2 component was accounted for by the recently developed Doniach-Sunjic-Shirley line [28] with asymmetry parameters optimized for highly defunctionalized samples and kept constant over the

whole series. Note that the photoemission intensity drop in the high BE tail (288-290 eV) is more pronounced for GO-Z, creating the shape of a small step, while for GO-M, it is rather smooth, demonstrating higher content of carboxyl groups in GO-Z. This tendency is preserved even for the films irradiated with high ion fluences and is in agreement with compositional differences between the films as deduced from the FT-IR data (Fig. 4b and Supplementary Fig. 8). The intensities of components due to oxygen functionalities decrease with increasing ion fluence, eventually reaching residual levels at high fluences.

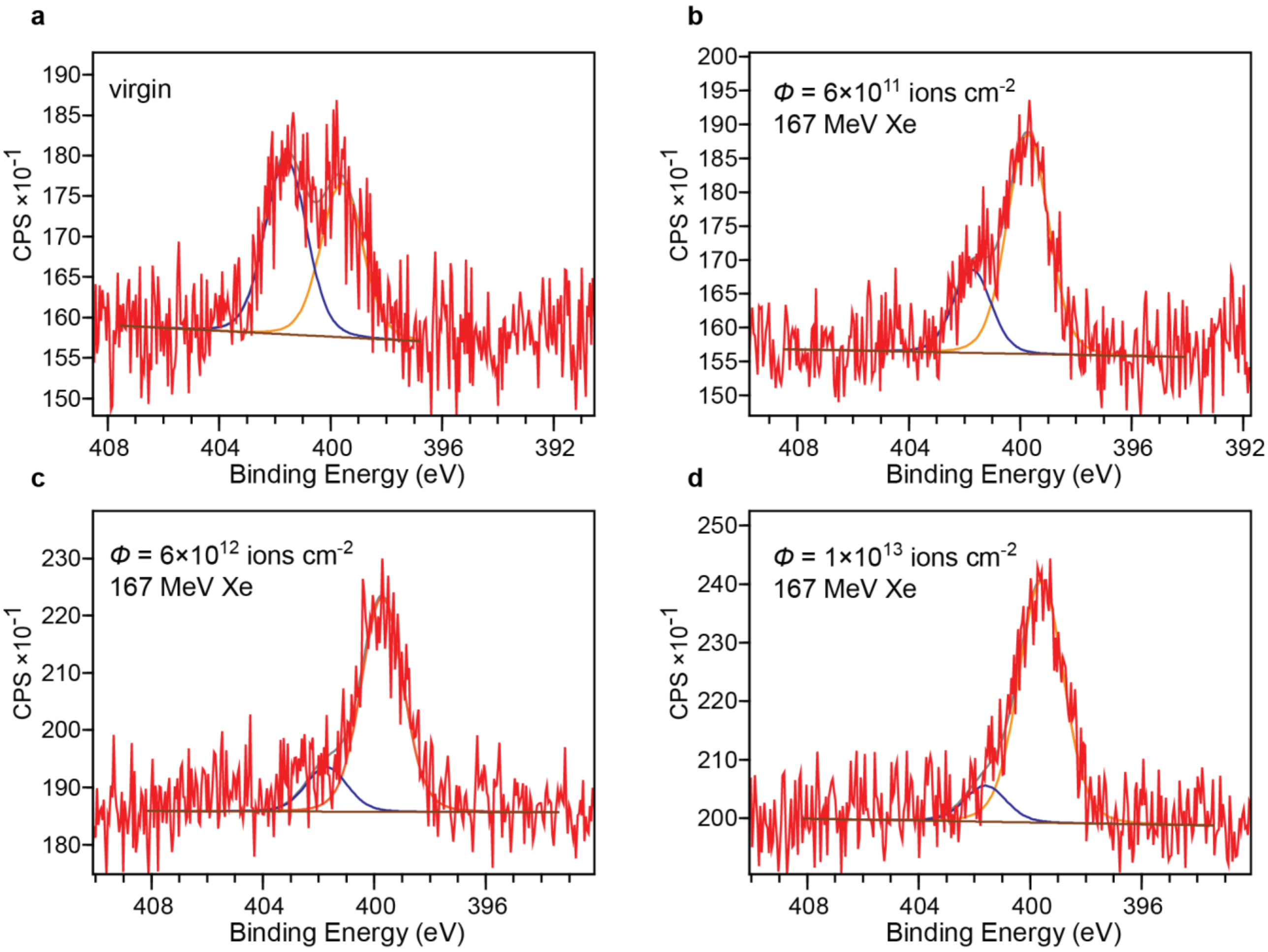

Supplementary Fig. 10: Evolution of the N 1*s* spectra of GO-Z films irradiated with different fluences of 167 MeV Xe ions. a The spectrum of the initial film, with two major components of comparable intensity (0.9:1) centered at 401.6 and 400 eV, is similar to those typically reported for GO obtained by the Hummers method [29, 30]. These components are tentatively ascribed to graphitic (NQ) and pyrrolic (N-5) nitrogen forms [31], but a reasonable attribution also assumes coincidental contaminants related to Hummers synthesis [29, 30]. **b-d** With increasing ion dose, the 401.6 eV (NQ) component is almost completely eliminated; at the same time, the N content increases from ca. 1 at.% (N/C ratio of 0.013) for the initial sample to ~ 1.6 at.% (N/C of 0.018) for films irradiated to fluences of $(0.6\text{-}1)\cdot 10^{13}$ ions/cm^2. **d** For the high-dose irradiated sample, the major component in the N 1*s* spectrum is the peak located at ~ 399 eV; the increase in the total N content indicates a contribution from nitrile nitrogen (C≡N), which shows a very similar binding energy value (~399 eV).

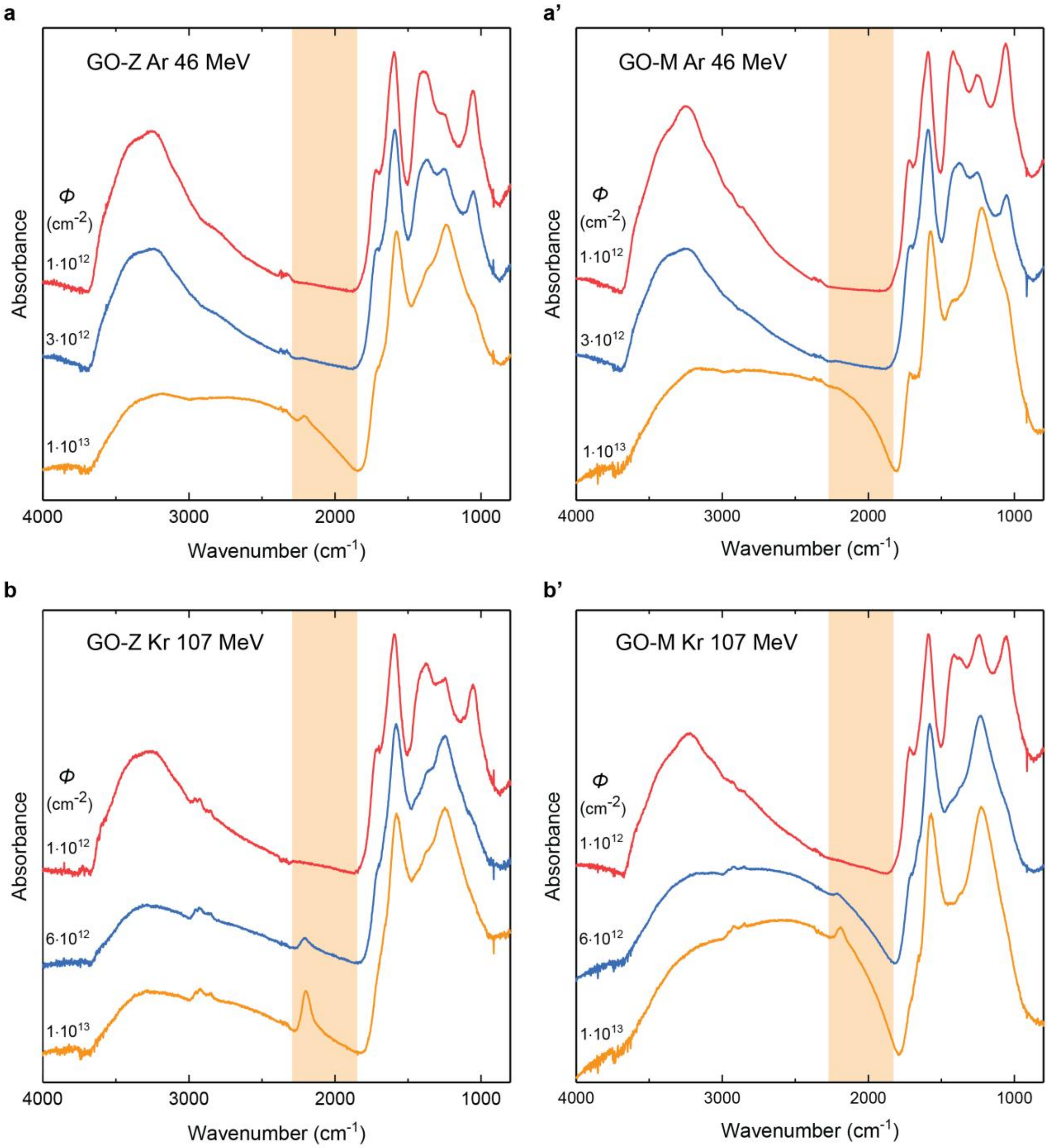

Supplementary Fig. 11: FT-IR spectra of GO films irradiated with 107 MeV Kr and 46 MeV Ar ions. a, a' GO-Z and GO-M films irradiated with Ar ions. **b, b'** GO-Z and GO-M films irradiated with Kr ions. The spectral region characteristic of conjugated C≡C bond stretching is highlighted in orange.

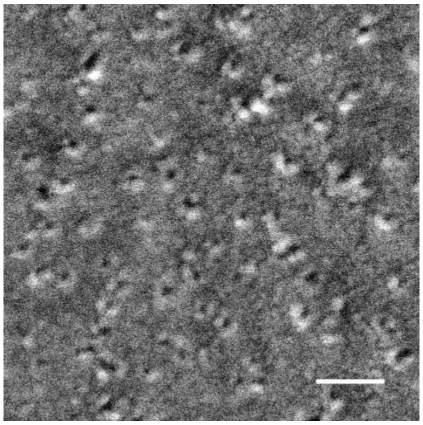

Supplementary Fig. 12: SEM image of the surface of a GO-M film irradiated with 710 MeV Bi ions. The fluence of Bi ions was $1 \cdot 10^{11}$ ions/cm^2, and the specimen was sputter coated with Pt/Pd prior to SEM investigation. Note that the created pores are not as pronounced as in the case of the GO-Z specimen irradiated under analogous conditions (Fig. 2c). The scale bar is 50 nm.

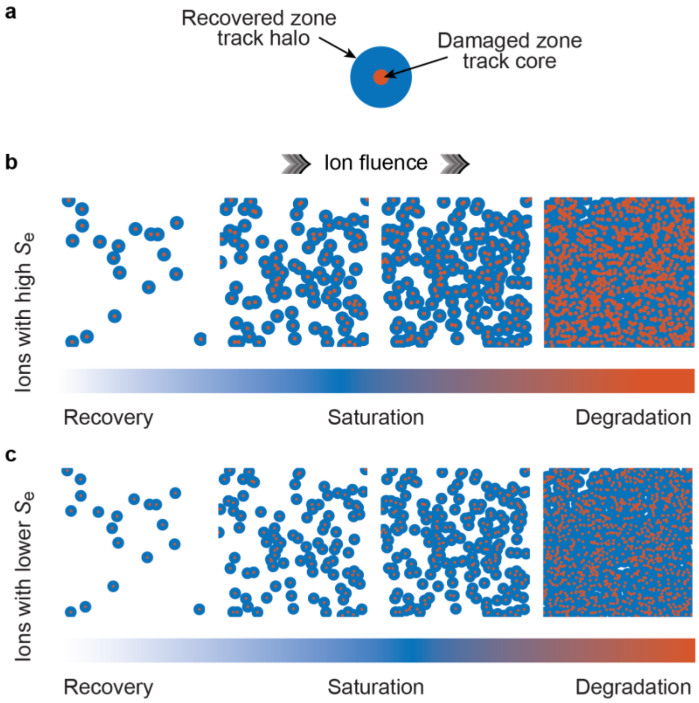

Supplementary Fig. 13: Stochastic core-halo model for damage/order evolution in SHI-irradiated GO. After ion impact, the temporary temperature increase is the highest in the track core (i.e., cylindrical region closest to the ion trajectory) and can be sufficient to cause damage or create nanoholes. In the track periphery (so-called track halo), the lattice heating is not as strong and leads to a partial defunctionalization of GO. The last process is similar to well-studied thermal defunctionalization (i.e, reduction), in which a partial restoration of graphitic structure occurs, except that it takes place locally, in the frontier region of the nanohole (high-S_e ions) or inside nanosized spots (low-S_e ions). **a** In the model,

we assume that (i) the damage and recovery are uniform and occur within the track core and track halo zone, respectively, and (ii) the radii of the track core and track halo are proportional to the damage and recovery efficiencies. The transport properties are improved in the recovered zones, and they are degraded in the damaged zones. **b** With increasing ion fluence, the recovered regions start to overlap; at high fluences, the damaged regions dominate, leading to conductivity degradation. **c** With decreasing S_e, the damage is suppressed to a greater extent than the recovery. For low-S_e ions, a saturation phase occurs at higher fluences, and the maximum achievable conductivity and extent of structural ordering are higher.

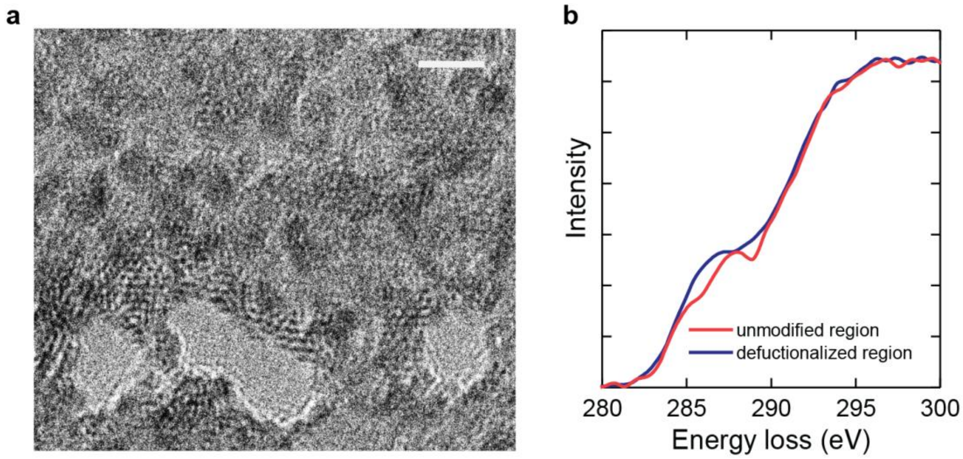

Supplementary Fig. 14: Analysis of SHI irradiation-induced structural and chemical transformation of GO by TEM and EELS. a TEM image of a suspended thin GO film irradiated with 710 MeV Bi ions at high magnification. The scale bar is 5 nm. Note that the frontier regions of densely spaced nanoholes are structurally modified. Because of the hybridization change from sp^3 to sp^2, these regions are flatter, with an orientation normal to the incident electron beam, allowing better imaging of the carbon planes. For unmodified GO (top part of the image), the clusters with well-visible graphitic planes are randomly distributed and are much smaller in size. **b** EELS spectra of the carbon K-edge acquired on unmodified and defunctionalized regions of the specimen. The following features due to π^* transitions are distinguishable: the peak at 287-288 eV is attributed to epoxy groups and chemisorbed water [32, 33, 34], and that of hydroxyl groups (~285.5 eV) forms a shoulder on the ~285 eV feature of sp^2 C. The feature at ~292.5 eV corresponds to the $1s$-σ^* transition. In the spectrum measured over a defunctionalized region, the peak at ~ 288 eV is less pronounced, and that of sp^2 C increases, indicating partial recovery of the graphitic structure around the nanoholes.

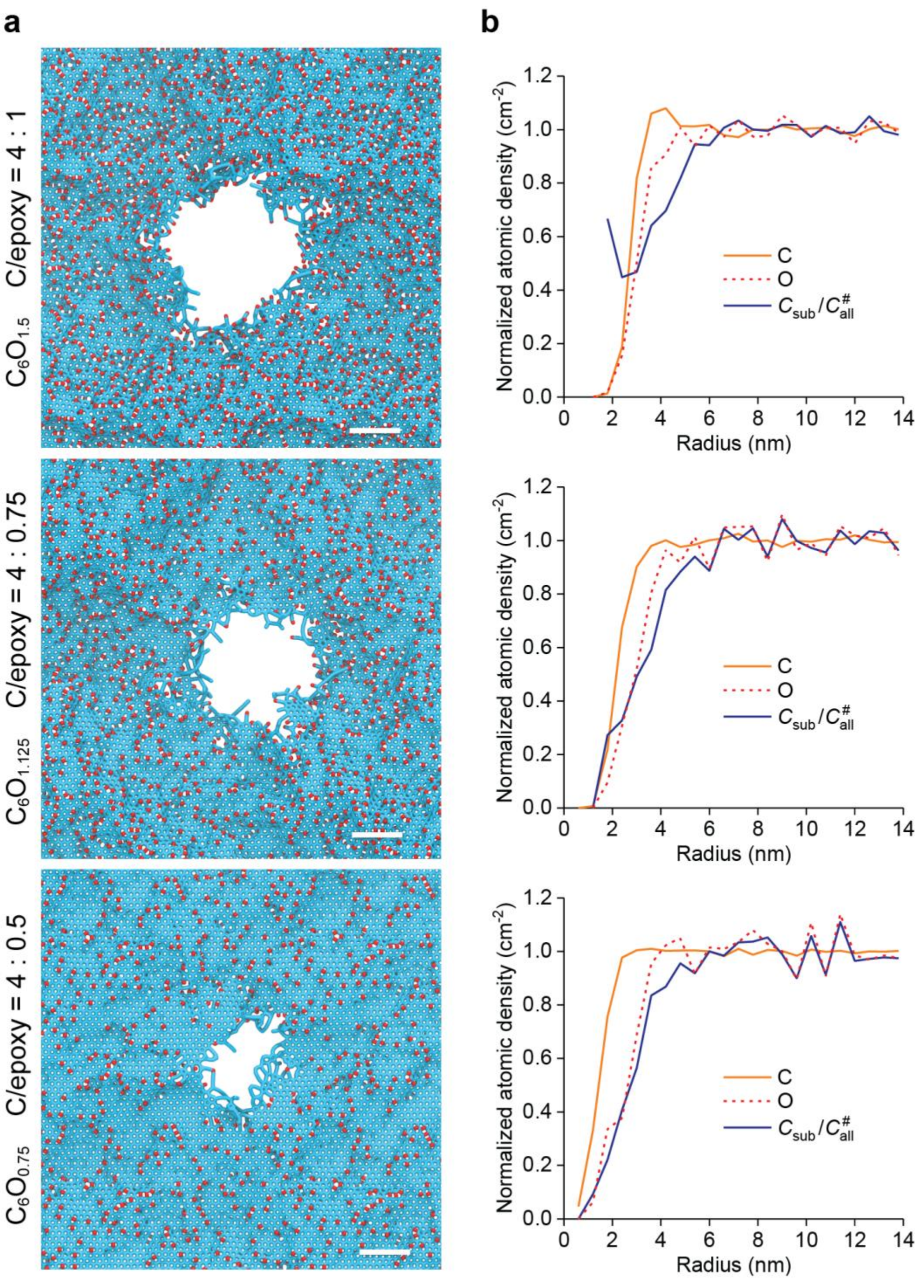

Supplementary Fig. 15: MC-RxMD simulations of 167 MeV Xe ion passage through single-layer model GO structures containing only epoxy groups. a Final structures 0.1 ns after projectile passage; the scale bar is 2 nm. **b** Respective radial distributions of normalized planar atomic densities and normalized local substitution degrees ($C_{sub}/C^{\#}_{all}$). Source data are provided as a Source Data file.

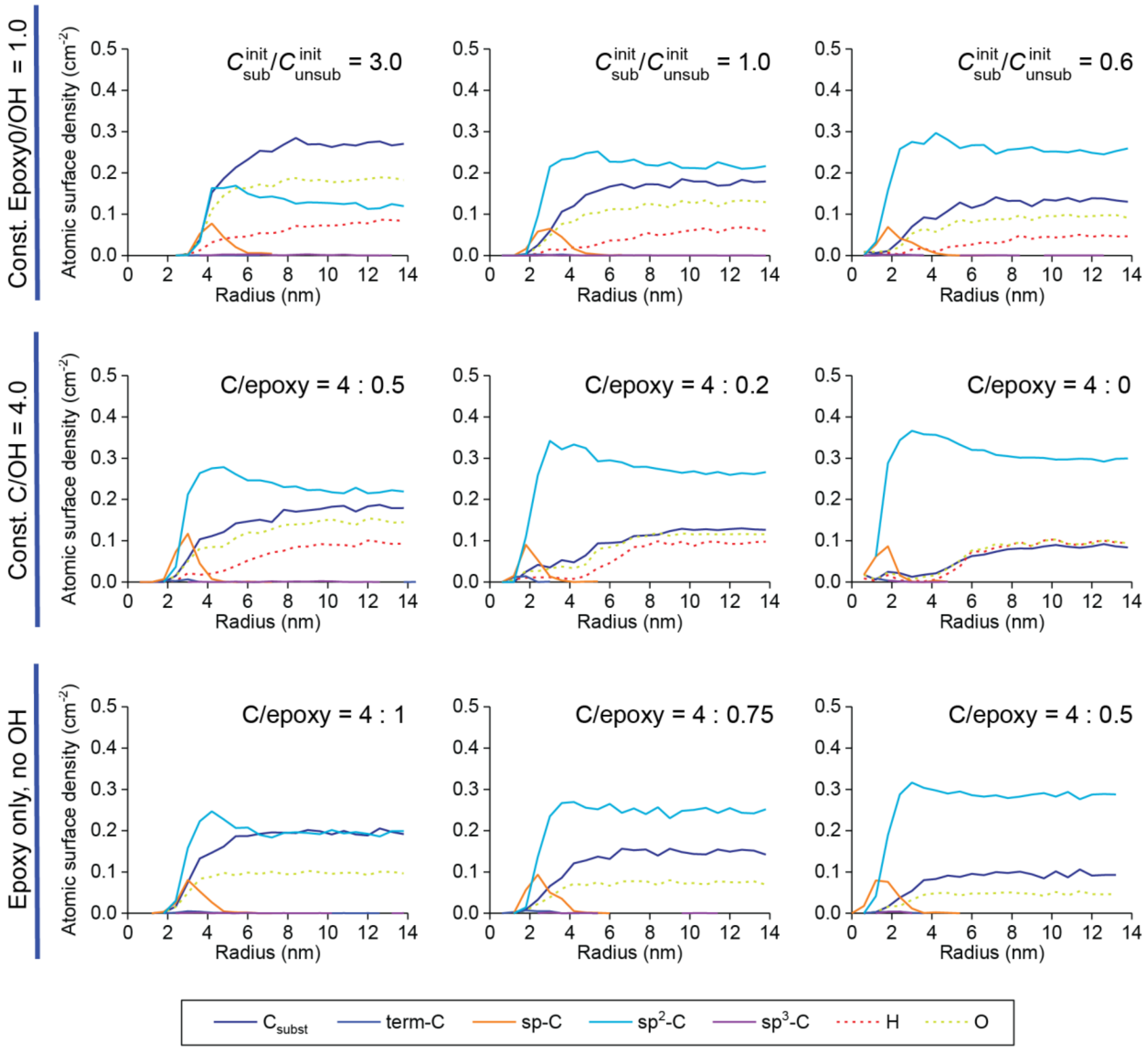

Supplementary Fig. 16: Radial distributions of the planar atomic densities of carbon atoms subdivided according to their type. C_{subst} denotes C atoms bonded to at least one heteroatom. Unsubstituted C atoms are categorized by their valence (i.e., terminal and *sp*-, sp^2-, and sp^3-hybridized). The radial distributions of H and O (dashed lines) are included for comparison. Source data are provided as a Source Data file.

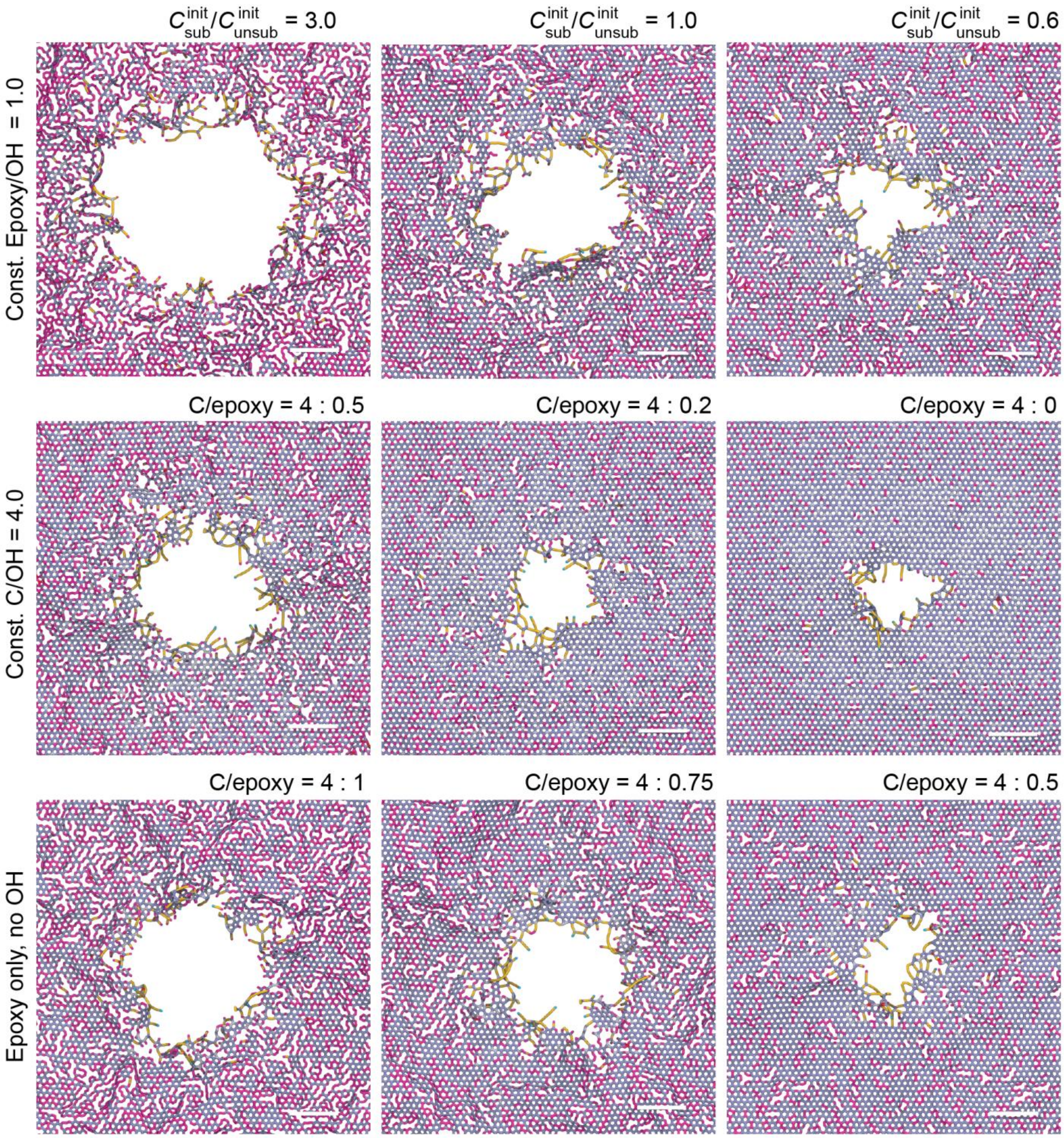

Supplementary Fig. 17: Carbon backbones of the GO structures after passage of a single 167 MeV Xe ion. Orthographic projections, scale bars are 2 nm. C atoms bonded to heteroatoms are colored in magenta. The unsubstituted C atoms are colored according to their valence: red for sp^3-C, cyan for terminal-C, orange for *sp*-C, dark gray for sp^2-C atoms with unchanged hybridization compared to the initial structure, and light gray for recovered sp^2-C.

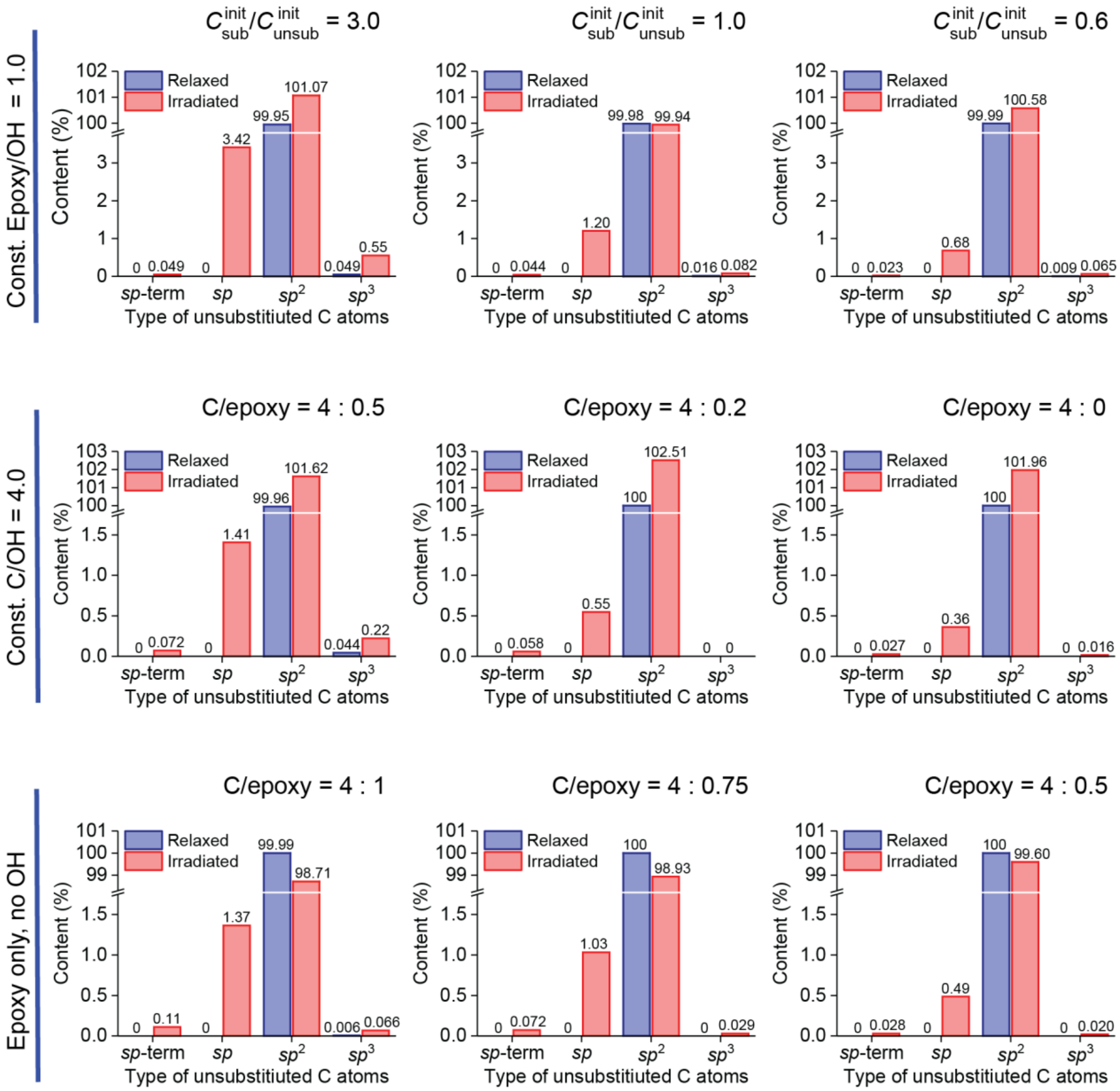

Supplementary Fig. 18: Distributions of unsubstituted carbon atoms for relaxed GO supercells and after the passage of a single 167 MeV Xe ion. For each GO model, both histograms were normalized to the number of unsubstituted C atoms in the relaxed cell. Source data are provided as a Source Data file.

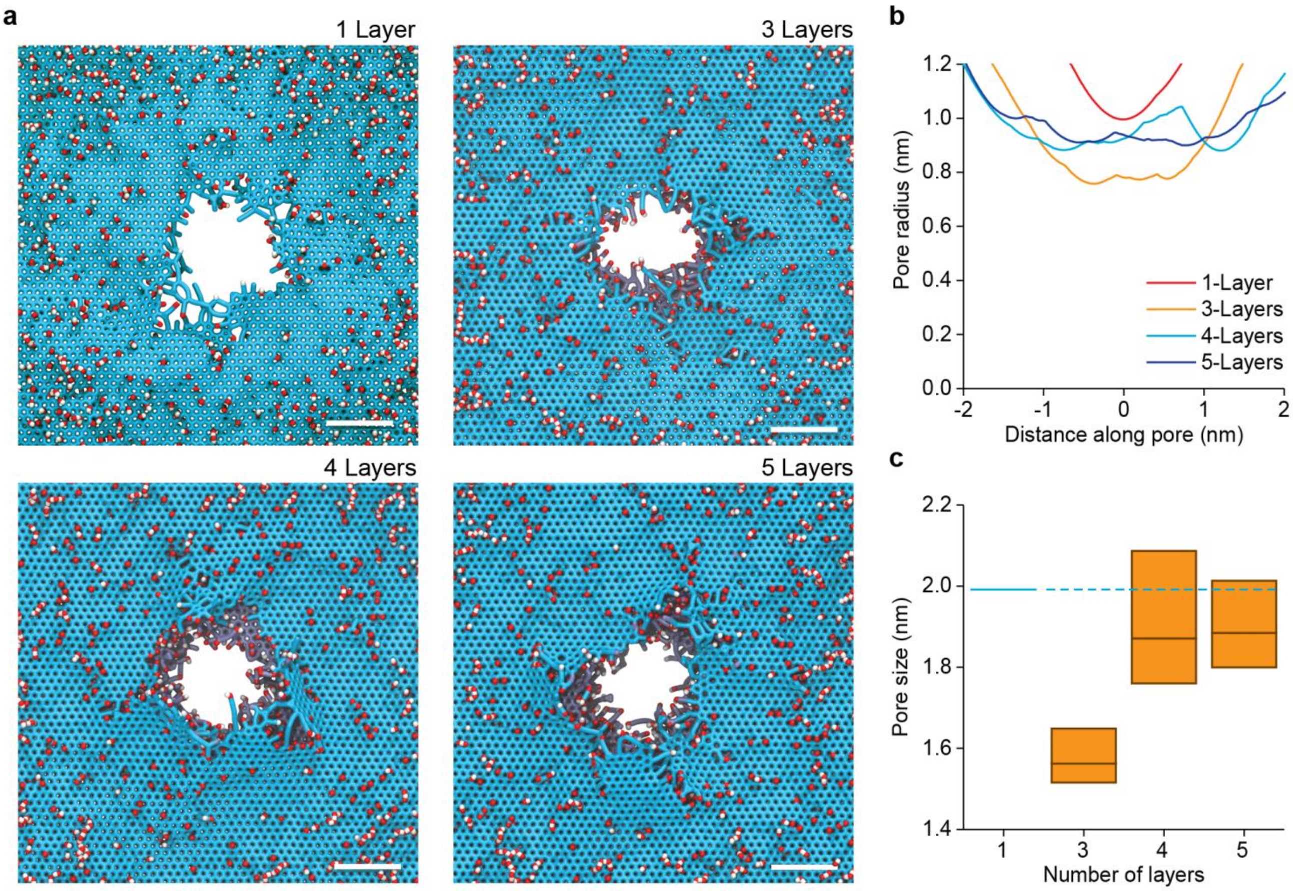

Supplementary Fig. 19: Creation of nanoholes in GO films consisting of different numbers of layers. For the simulations, the initial composition of C:H:O 6:1.5:1.8 was chosen, and the impact of the 167 MeV Xe ion was modeled. **a-**Final structures 0.1 ns after projectile passage of single-, three-, four-, and five-layer GO. The scale bars are 2 nm; for better visualization of differences between inner and outer layers, the atoms of the internal layers are colored in ice blue. Note that for single-layer GO, the thorns appearing at the nanohole edge closely resemble those visible in the experimental TEM image (Fig. 1a). For few-layer GO, there are still some cracks in the outer layer, but the nanoholes in the internal layers have an almost perfect round-shaped geometry. **b** Nanopore profiles obtained with HOLE code employing AMBER van der Waals radii. **c** Corresponding min-max ranges of the nanopore radii for GO structures consisting of different numbers of layers. Source data are provided as a Source Data file.

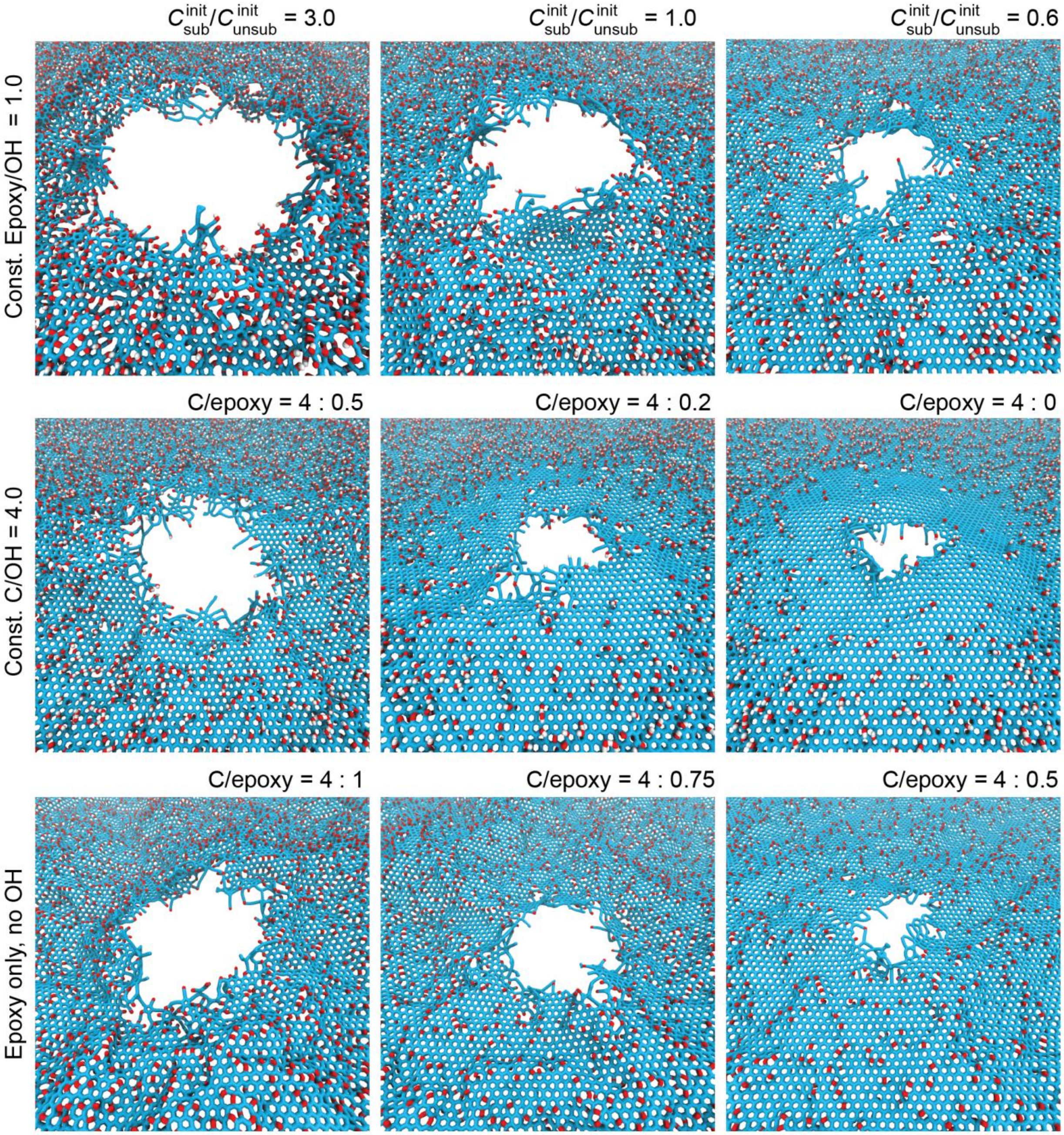

Supplementary Fig. 20: GO structures after passage of a single 167 MeV Xe ion. Zoomed-in perspective views at a 30° tilt angle. Note that for structures prone to irradiation-induced defunctionalization (e.g., C/OH = 4, C/epoxy = 4:0.2 and C/OH = 4, C/epoxy = 4:0), the recovered regions become curved because of the in-plane lattice expansion resulting from the sp^3-to-sp^2 transformation.

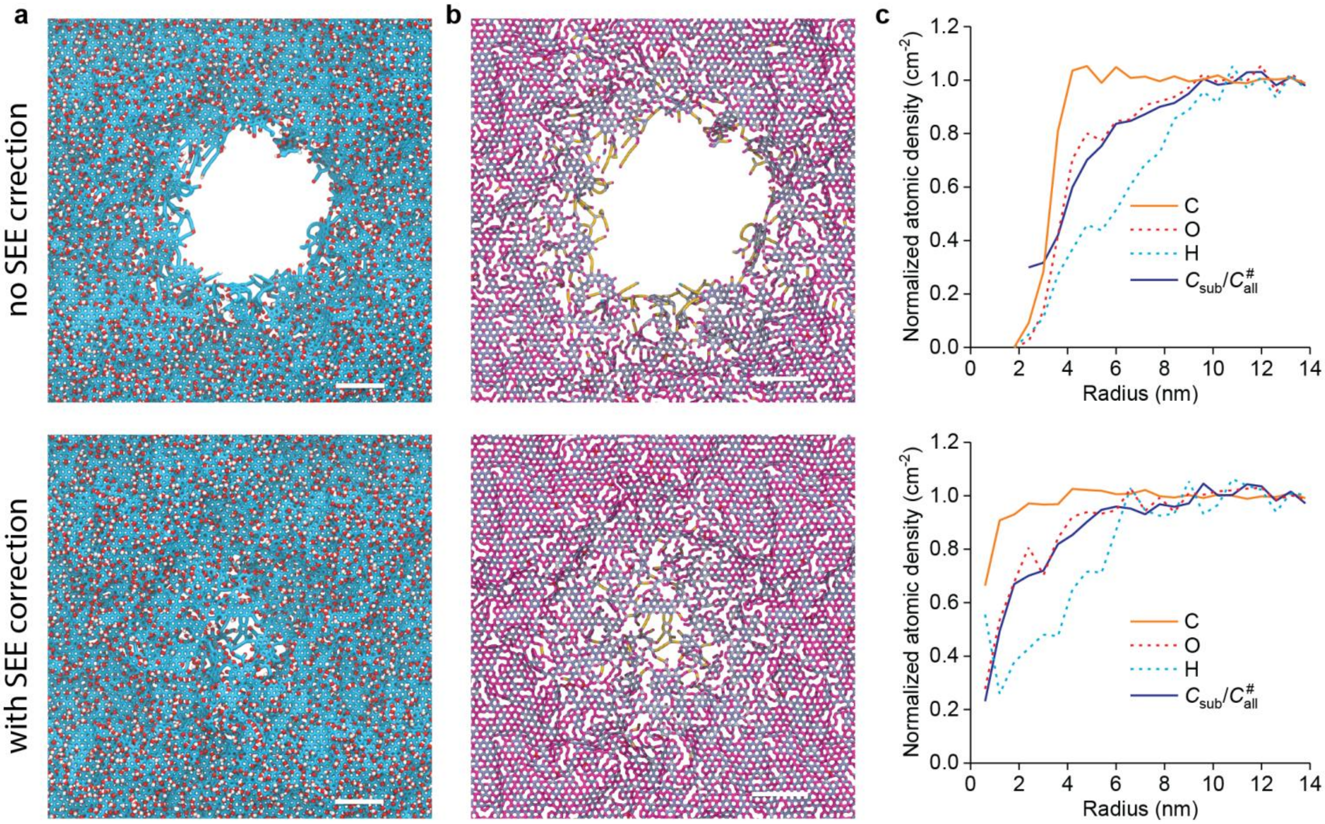

Supplementary Fig. 21. Effect of secondary electron emission on the extent of structural modification of single-layer GO by SHI irradiation. Comparison of SEE-uncorrected **top** and -corrected **bottom** MC-RxMD simulation results for a single 167 MeV Xe ion-irradiated GO model structure with an initial C/O ratio of 2.5 and an OH/epoxy composition typical of Hummers synthesis (2:1). The reduction in energy deposition due to SEE was assumed to be the same as that estimated for single-layer graphene (50%). **a** Final structures and **b** carbon backbones 0.1 ns after projectile passage (the scale bars are 2 nm; for the carbon atom color coding, refer to the caption of Supplementary Fig. 17). **c** Respective radial distributions of normalized planar atomic densities and normalized local $C_{\mathrm{sub}}/C_{\mathrm{all}}$ ratios. Source data are provided as a Source Data file.

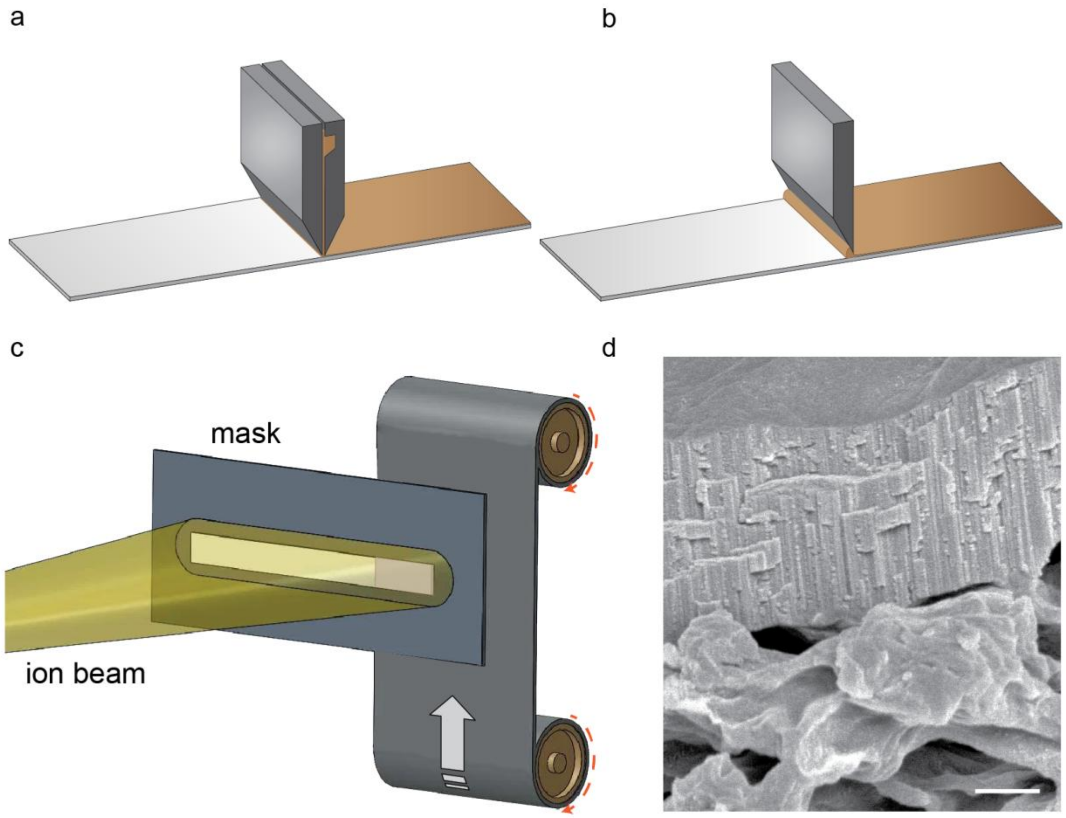

Supplementary Fig. 22: Schematic representation of a proposed scalable roll-to-roll fabrication method of hybrid GO-based track membranes consisting of an SHI-patterned GO thin film (selective layer) supported on a porous polymer substrate (passive layer). a,**b** Coating of the substrate with the GO layer using a scalable and roll-to-roll compatible processing method, such as **a** doctor blade coating or **b** slot-die coating. Bar coating, or its recent modification based on a Mayer rod, can be used as well. In these techniques, the nematic GO dispersion is transformed into aligned GO layers, providing exceptional mechanical properties of the coated films [35]. **c** Heavy-ion irradiation of the composite films using a roll-to-roll chamber facility. **d** Cross-sectional SEM image of an exemplary hybrid GO-based track membrane obtained in a pilot lab-scale process employing vacuum filtration of GO onto a 0.2 µm porous nylon 66 film. Note that in the nylon support, ion-track pores were not formed, and the inherent pores are large enough to provide undisturbed flow of the solution in separation-oriented applications. The scale bar is 0.5 µm

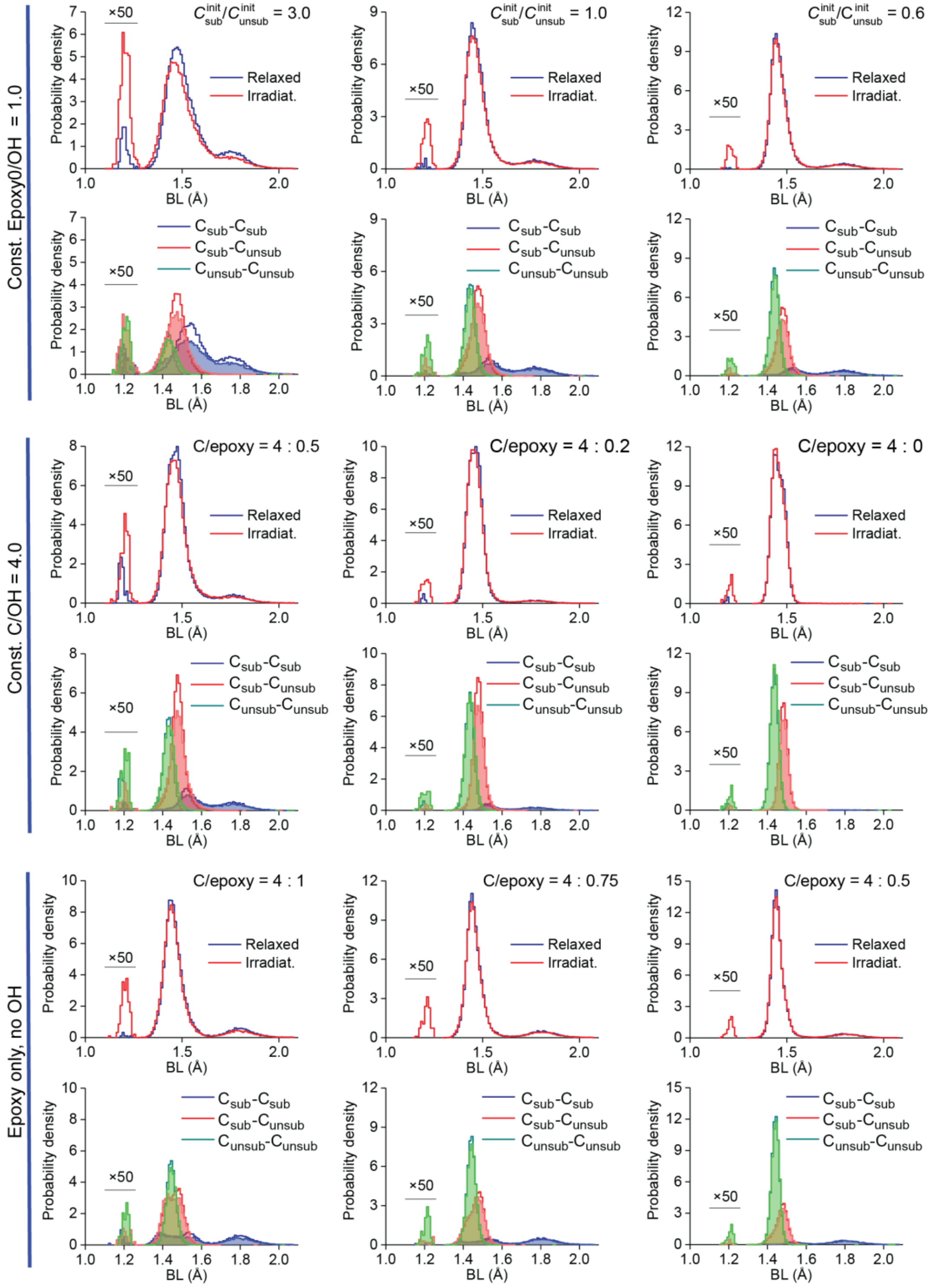

Supplementary Fig. 23: C-C bond length distributions for relaxed GO supercells and after the passage of a single 167 MeV Xe ion. top rows Total distributions, **bottom rows** partial distributions. For each GO model, each histogram was normalized to the total number of C-C bonds in a respective relaxed cell. The partial distributions for ion-irradiated GO cells are represented with color-filled bars, and the bars for relaxed cells are unfilled.

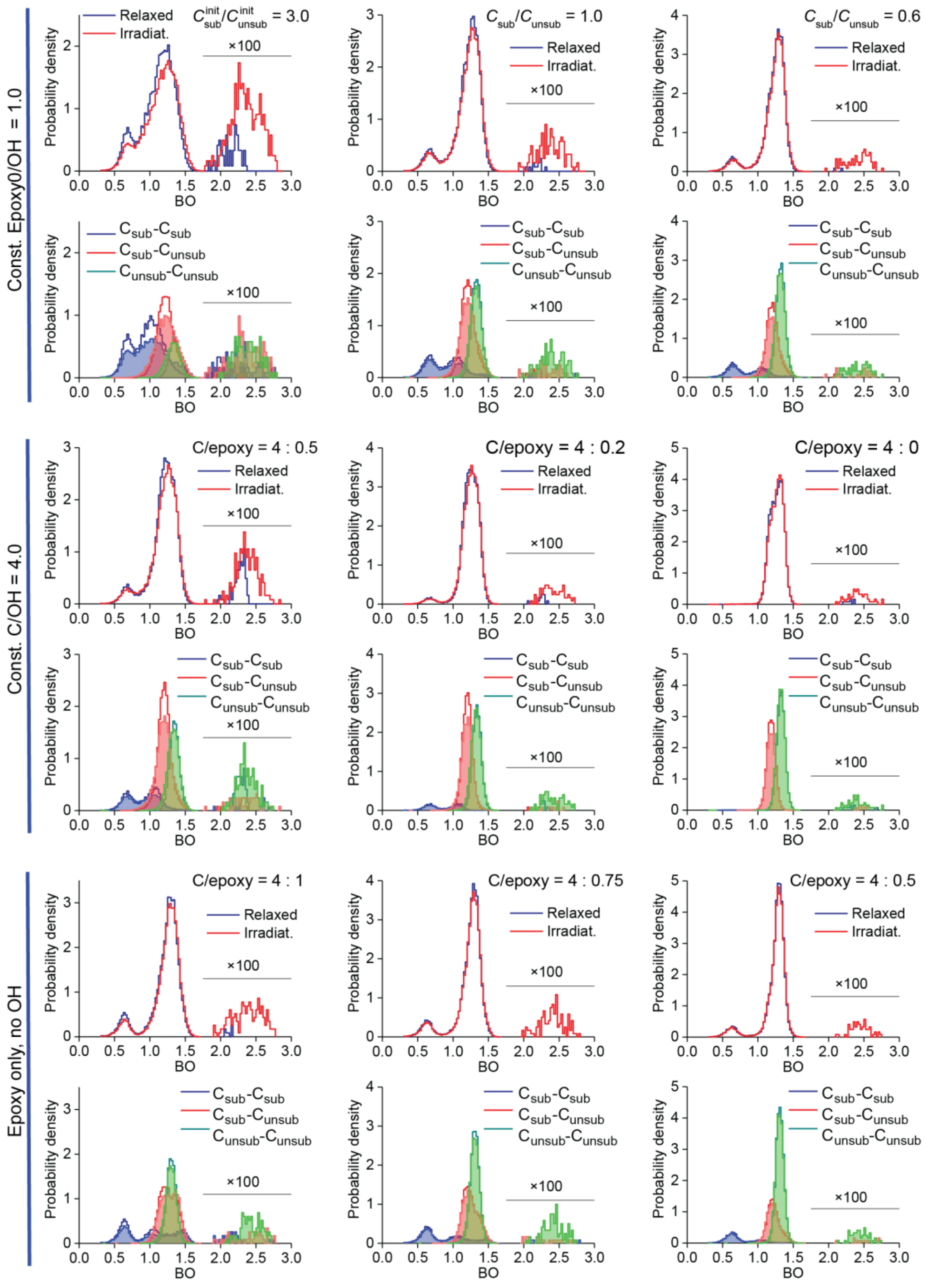

Supplementary Fig. 24: C-C bond order distributions for relaxed GO supercells and after the passage of a single 167 MeV Xe ion. top rows Total distributions, **bottom rows** partial distributions. For each GO model, each histogram was normalized to the total number of C-C bonds in a respective relaxed cell. The partial distributions for ion-irradiated GO cells are represented with color-filled bars, and the bars for relaxed cells are unfilled.

Supplementary tables

Supplementary Table 1. Accelerated ions, their energies, and their electronic stopping powers in GO.

Ion	Energy (MeV)	*S*e (keV/nm)[a]
Bi	710	22.1
Xe	167	14.3
Kr	107	9.4
V	61	6.2
Ar	40	4.7

a – calculated with SRIM-2013 code assuming a C:H:O composition of GO of 6:1.2:2.4, i.e., with the same C/O ratio as that of our samples (2.5) and an OH/epoxy ratio typical of Hummers synthesis (2:1).

Supplementary Table 2. Additional compositional parameters for single-layer GO models studied with MC-RxMD.

Formula	C:epoxy:OH	Initial	Relaxed	After ion passage	
		C_{sub}/C_{unsub}	C_{sub}/C_{unsub}	C_{sub}/C_{unsub}	C_{rem}/C_{all}
Constant epoxy/OH ratio of 1.0, varied substitution ratio					
$C_6H_{1.5}O_3$	4:1:1	3.00	2.28	1.99	0.042
$C_6H_{1.0}O_2$	4:0.66:0.66	1.00	0.821	0.766	0.018
$C_6H_{0.75}O_{1.5}$	4:0.5:0.5	0.60	0.538	0.504	0.010
Constant C/OH ratio of 4.0, varied C/epoxy ratio					
$C_6H_{1.5}O_{2.25}$	4:0.5:1	1.00	0.837	0.744	0.019
$C_6H_{1.5}O_{1.8}$	4:0.2:1	0.54	0.489	0.432	0.0085
$C_6H_{1.5}O_{1.5}$	4:0:1	0.33	0.291	0.257	0.0029
Only epoxy groups (no OH)					
$C_6O_{1.5}$	4:1:0	1.00	1.00	0.955	0.021
$C_6O_{1.125}$	4:0.75:0	0.60	0.60	0.576	0.015
$C_6O_{0.75}$	4:0.5:0	0.33	0.33	0.325	0.006

C_{rem}/C_{all} represents the ratio of C atoms removed from the GO layer to C atoms bonded to the GO layer; C_{sub}/C_{unsub} denotes the ratio of substituted C atoms to unsubstituted C atoms in the GO layer.

Supplementary Table 3: Coefficients of the inverse complex dielectric function of GO in the form of the sum of oscillator functions. E_{0i}, A_i, and γ_i are coefficients of Eq. (S7) used for reconstruction of the loss function. *f*-sum is the number of electrons (N_e) on the atomic shells. The values from [36] are shown in brackets for comparison.

Name	E_{0i}	A_i	γ_i	*f*-sum (Number of electrons per formula unit)
Valence Band	1.8	-0.15	1	15.01432 (15)
	3	0.7	4	
	7	5	1	
	10	8	3	
	19.5	45	6	
	28	524	11.5	
K-shell of C	290	214	230	3.98453 (4)
K-shell of O	500	128	300	2.00759 (2)
Total:				21.00644 (21)

Supplementary References

1. Härdle & Simar, L., *Applied Multivariate Statistical Analysis* (Springer, 2007).
2. Scott, D. W., On optimal and data-based histograms. *Biometrika* **66**, 605-610 (1979).
3. Liscio, A. *et al.*, Evolution of the size and shape of 2D nanosheets during ultrasonic fragmentation. *2D Materials* **4**, 025017 (2017).
4. Mravik, Ž. & Jovanović, Z., Analysis of surface oxygen groups of thermally reduced graphene oxide via temperature programmed desorption method. *Tehnika - Novi Materiali* **73**, 186-191 (2018).
5. Zhang, L. *et al.*, Size-controlled synthesis of graphene oxide sheets on a large scale using chemical exfoliation. *Carbon* **47**, 3365-3368 (2009).
6. Wychowaniec, J. K. *et al.*, Designing Peptide/Graphene Hybrid Hydrogels through Fine-Tuning of Molecular Interactions. *Biomacromolecules* **19**, 2731-2741 (2018).
7. Paton, K. R. *et al.*, Scalable production of large quantities of defect-free few-layer graphene by shear exfoliation in liquids. *Nature Materials* **13**, 624-630 (2014).
8. Gacka, E., Majchrzycki, Ł., Marciniak, B. & Lewandowska-Andralojc, A., Effect of graphene oxide flakes size and number of layers on photocatalytic hydrogen production. *Scientifc Reports* **11** (2021).
9. Pines, D., *Elementary Excitations In Solids* (W.A. Benjamin Inc., New York – Amsterdam, 1963).
10. Medvedev, N. A., Rymzhanov, R. A. & Volkov, A. E., Time-resolved electron kinetics in swift heavy ion irradiated solids. *Journal of Physics D: Applied Physics* **48**, 355303 (2015).
11. Rymzhanov, R. A., Medvedev, N. A. & Volkov, A. E., Effects of model approximations for electron, hole, and photon transport in swift heavy ion tracks. *Nucl. Instruments Methods Phys. Res. Sect. B* **388**, 41-52 (2016).
12. Ritchie, R. H. & Howie, A., Electron excitation and the optical potential in electron microscopy. *Philos. Mag.* **36**, 463-481 (1977).
13. Gangwar, P., Singh, S. & Khare, N., Study of optical properties of graphene oxide and its derivatives using spectroscopic ellipsometry. *Applied Physics A* **124** (2018).
14. Schöche, S. *et al.*, Optical properties of graphene oxide and reduced graphene oxide determined by spectroscopic ellipsometry. *Applied Surface Science* **421**, 778-782 (2017).
15. Henke, B. L., Gullikson, E. M. & Davis, J. C., X-Ray Interactions: Photoabsorption, Scattering, Transmission, and Reflection at E = 50-30,000 eV, Z = 1-92. *Atomic Data and Nuclear Data Tables* **54**, 181-342 (1993).
16. in *X-ray interactions with matter.*
17. Palik, E. D., *Optical Parameters for the Materials in HOC I, HOC II, and HOC III* (Academic Press, 1998).
18. Mott, N. & Massey, H. S. W., *The Theory of Atomic Collisions, 2nd ed.* (Oxford University Press, London, 1949).
19. Jenkins, T. M., Nelson, W. R. & Rindi, A. eds., *Monte Carlo Transport of Electrons and Photons* (Springer US, Boston, 1988).
20. Rymzhanov, R. A., Medvedev, N. & Volkov, A. E., Damage threshold and structure of swift heavy ion tracks in Al_2O_3. *Journal of Physics D: Applied Physics* **50**, 475301 (2017).

21. Plimpton, S., Fast Parallel Algorithms for Short-Range Molecular Dynamics. *Computational Physics* **117**, 1-19 (1995).
22. Srinivasan, S. G. & van Duin, A. C. T., Molecular-Dynamics-Based Study of the Collisions of Hyperthermal Atomic Oxygen with Graphene Using the ReaxFF Reactive Force Field. *Journal of physical chemistry A* **115**, 13269-13280 (2011).
23. Li, Z., Zhang, W., Luo, Y., Yang, J. & Hou, J. G., How Graphene Is Cut upon Oxidation? *Journal of the American Chemical Society* **131**, 6320-6321 (2009).
24. Luo, H., Auchterlonie, G. & Zou, J., A thermodynamic structural model of graphene oxide. *Journal of Applied Physics* **122**, 145101 (2017).
25. Sun, Y., Tang, X., Bao, H., Yang, Z. & Ma, F., The effects of hydroxide and epoxide functional groups on the mechanical properties of graphene oxide and its failure mechanism by molecular dynamics simulations. *RSC Adv.* **10**, 29610-29617 (2020).
26. Berendsen, H. J. C., Postma, J. P. M., van Gunsteren, W. F., DiNola, A. & Haak, J. R., Molecular dynamics with coupling to an external bath. *Journal of Chemical Physics* **81**, 3684-3690 (1984).
27. Jovanovic, Z. *et al.*, The role of surface chemistry in the charge storage properties of graphene oxide. *Electrochimica Acta* **258**, 1228-1243 (2017).
28. Moeini, B. *et al.*, Definition of a new (Doniach-Sunjic-Shirley) peak shape for fitting asymmetric signals applied to reduced graphene oxide/graphene oxide XPS spectra. *Surf Interface Anal.* **54**, 67-77 (2022).
29. Chua, C. K. *et al.*, Chemical Preparation of Graphene Materials Results in Extensive Unintentional Doping with Heteroatoms and Metals. *Chemistry - A European Journal* **20**, 15760-15767 (2014).
30. Barbolina, I. *et al.*, Purity of graphene oxide determines its antibacterial activity. *2D Materials* **3**, 025025 (2016).
31. Pels, J. R., Kapteijn, F., Moulijn, J. A., Zhu, Q. & Thomas, K. M., Evolution of nitrogen functionalities in carbonaceous materials during pyrolysis. *Carbon* **33**, 1641-1653 (1995).
32. Pelaez-Fernandez, M., Bermejo, A., Benito, A. M., Maser, W. K. & Arenal, R., Detailed thermal reduction analyses of graphene oxide via in-situ TEM/EELS studies. *Carbon* **178**, 477-487 (2021).
33. D'Angelo, D. *et al.*, Electron energy-loss spectra of graphene oxide for the determination of oxygen functionalities. *Carbon* **93**, 1034-1041 (2015).
34. Hettler, S. *et al.*, In-situ reduction by Joule heating and measurement of electrical conductivity of graphene oxide in a transmission electron microscope. *2D Materials* **8**, 031001 (2021).
35. Akbari, A. *et al.*, Large-area graphene-based nanofiltration membranes by shear alignment of discotic nematic liquid crystals of graphene oxide. *Nature Communications* **7**, 1-12 (2016).
36. Lide, D. R. ed., *CRC Handbook of Chemistry and Physics, 84th Edition* (CRC Press LLC: Boca Raton, 2004).
