# Supplementary material for: From nanohole to ultralong straight nanochannel fabrication in graphene oxide with swift heavy ions
Source: Nat Commun. 2023 Feb 16;14:889. doi: 10.1038/s41467-023-36357-8 (PMC9935919; doi:10.1038/s41467-023-36357-8)
Supplement: Supplementary file 3 — Description of Additional Supplementary Files [file 41467_2023_36357_MOESM3_ESM.pdf]

Description of Additional Supplementary Files

File Name: Supplementary Movie 1
Description: Dynamics of nanopore formation in single layer GO ($C_6H_{1.5}O_{1.8}$, tilted perspective view). The first 5 ps of the animation is shown in slow motion.

File Name: Supplementary Movie 2
Description: Dynamics of nanopore formation in 3-layer GO ($C_6H_{1.5}O_{1.8}$, cross-sectional view).

File Name: Supplementary Movie 3
Description: Dynamics of nanopore formation in multilayer GO (10 layers, $C_6H_{1.2}O_{2.4}$, cross-sectional view). The first 5 ps of the animation is shown in slow motion.
